# A Bistable Gene Switch for Antibiotic Biosynthesis: The Butyrolactone Regulon in *Streptomyces coelicolor*

**DOI:** 10.1371/journal.pone.0002724

**Published:** 2008-07-16

**Authors:** Sarika Mehra, Salim Charaniya, Eriko Takano, Wei-Shou Hu

**Affiliations:** 1 Department of Chemical Engineering, Indian Institute of Technology Bombay, Powai, Mumbai, India; 2 Department of Chemical Engineering and Materials Science, University of Minnesota, Minneapolis, Minnesota, United States of America; 3 Department of Microbiology, University of Groningen, Groningen, The Netherlands; Center for Genomic Regulation, Spain

## Abstract

Many microorganisms, including bacteria of the class Streptomycetes, produce various secondary metabolites including antibiotics to gain a competitive advantage in their natural habitat. The production of these compounds is highly coordinated in a population to expedite accumulation to an effective concentration. Furthermore, as antibiotics are often toxic even to their producers, a coordinated production allows microbes to first arm themselves with a defense mechanism to resist their own antibiotics before production commences. One possible mechanism of coordination among individuals is through the production of signaling molecules. The γ-butyrolactone system in *Streptomyces coelicolor* is a model of such a signaling system for secondary metabolite production. The accumulation of these signaling molecules triggers antibiotic production in the population. A pair of repressor-amplifier proteins encoded by *scbA* and *scbR* mediates the production and action of one particular γ-butyrolactone, SCB1. Based on the proposed interactions of *scbA* and *scbR*, a mathematical model was constructed and used to explore the ability of this system to act as a robust genetic switch. Stability analysis shows that the butyrolactone system exhibits bistability and, in response to a threshold SCB1 concentration, can switch from an OFF state to an ON state corresponding to the activation of genes in the cryptic type I polyketide synthase gene cluster, which are responsible for production of the hypothetical polyketide. The switching time is inversely related to the inducer concentration above the threshold, such that short pulses of low inducer concentration cannot switch on the system, suggesting its possible role in noise filtering. In contrast, secondary metabolite production can be triggered rapidly in a population of cells producing the butyrolactone signal due to the presence of an amplification loop in the system. *S. coelicolor* was perturbed experimentally by varying concentrations of SCB1, and the model simulations match the experimental data well. Deciphering the complexity of this butyrolactone switch will provide valuable insights into how robust and efficient systems can be designed using “simple” two-protein networks.

## Introduction

Many microorganisms make antibiotics that confer a competitive advantage for their survival. Streptomycetes (genus *Streptomyces*) in particular produce nearly 70% of antibiotics in clinical use [1]. These are soil microorganisms, which in their natural habitat grow in small colonies in an environment where the physical and chemical conditions fluctuate constantly. The arsenal of antibiotics helps them compete with other organisms in the soil environment under stresses due to various adverse conditions. However, accumulation of antibiotics to an effective concentration takes time–a process that can be expedited if the entire population acts in a synchronized manner. As antibiotics are often toxic even to their producers, microbes first express a defense mechanism to resist their own antibiotics before turning these weapons against others. Coordination within members of a population is critical because uncoordinated antibiotic production by some members could be fatal for others of the same species in the population which fail to equip themselves with a defense mechanism.

In a natural environment the transition from a favorable state to an adverse state may not be as defined as under controlled conditions in the laboratory. The environmental cues or signals which indicate impending adverse conditions are likely to be riddled with noise. Differentiating between these cues and noises is vital for a coordinate response and thereby the survival of the population. One possible mechanism of noise filtering is through a mechanism of “voting” by members in the population. If a large number of surrounding neighbors are positively responding to the cue and begin to change their physiological state, then the cue is more likely to be true. Conversely, if the number is small, then it is highly probable that the cue is false; under such circumstance those which have responded should return to the original state and the responsive action taken by them will subside. One mechanism of voting is by sensing and producing of signaling molecules. A rapid accumulation of signaling molecules is an indication that a large number of neighbors are producing the signaling molecule in response to the environmental cue; this is likely to indicate the right moment to produce antibiotics.

γ-butyrolactone are a class of signaling molecules (for review, see [2]) that were first ever identified from a microbe. Other classes of signaling molecules have now been identified in many bacteria including the homoserine lactones, which regulate many physiological aspects like biofilm formation and have been shown to be important in virulence in *Pseudomonas aeruginosa* (reviewed in [3]). The existence of these multiple signaling molecules that are widespread throughout the bacterial kingdom suggests the importance of systems using such mechanism. In *Streptomyces griseus* a γ-butyrolactone that was first identified called the A-factor, controls both antibiotic production and sporulation [4]. Since the discovery of A-factor, γ-butyrolactones have been identified in many *Streptomyces* species [2]. *Streptomyces coelicolor*, the most widely studied model organism of the *Streptomyces* genus, produces at least three different γ-butyrolactones. Among them, SCB1 is the best characterized γ-butyrolactone [5]. The addition of SCB1 to an agar culture elicited localized antibiotic production in *S. coelicolor*. Similar effects of γ-butyrolactones on antibiotics production have been observed in virginiamycin production in *Streptomyces virginiae* [6], showdomycin and minimycin production in *Streptomcyes lavendulae* FRI-5 [7], [8], and in clavulanic acid production in *Streptomyces clavuligerus* [9]. However, unlike A-factor in *S. griseus*, most of these γ-butyrolactones are involved only in the regulation of antibiotic production and related aspects of secondary metabolism and are not essential for morphological development.

A gene pair, *scbA* and *scbR*, is involved in the regulation of the synthesis of γ-butyrolactone, SCB1, in *S. coelicolor*. A transient surge of the transcript of the two genes forebodes the rapid increase of SCB1 and the synthesis of the antibiotics they regulate. Homologues of ScbA and ScbR have been found in *Streptomyces virginiae*, [6] *Streptomyces fradiae*, [10], [11] *Streptomyces lavendulae* [12], *Streptomyces pristinaespiralis* [13], *Streptomyces clavuligerus* [9], *Streptomyces lividans* [14] and *Kitasatospora setae*, a non-streptomyces species of actinomycetes [15], though the role of these homologues differs or is not known in some species. This two-gene γ-butyrolactone signaling system entails a cytoplasmic receptor protein (ScbR) and an amplifier protein (ScbA). The receptor protein ScbR has a γ-butyrolactone-binding domain at the *C*-terminal and a characteristic DNA binding helix-turn-helix motif at its *N*-terminal [16], [17]. ScbR directly binds to the promoter region of the gene encoding the pathway-specific regulator (CpkO) of cryptic type I polyketide synthase gene cluster (*cpk*) and represses its expression [18]. Binding of SCB1 to ScbR abolishes the repression of *cpkO* and allows the expression of genes *in cpk* cluster. These results suggest that sufficient levels of free ScbR are present during early exponential phase to suppress expression of genes in *cpk* cluster. Also, recent studies have strongly suggested that the amplifier protein ScbA, a homologue of AfsA, a key enzyme in the biosynthesis of A-factor [19], is likely to play an enzymatic role in SCB1 synthesis in addition to a possible regulatory role [20].

**Figure 1:**
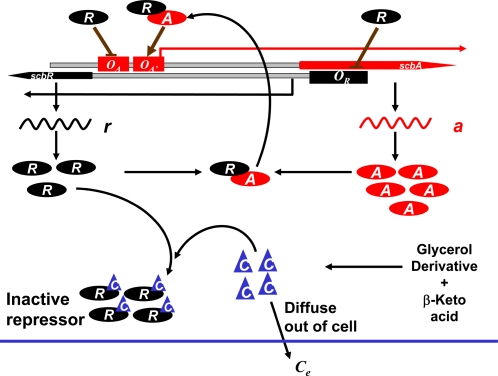
Schematic diagram of the ScbA/ScbR system. The two DNA strands are shown in black. Gene *scbA* is present on the positive strand (shown in red) whereas *scbR* is present on the opposite strand (shown in black). Protein ScbR (*R*) can bind to operator site *O_R_* to repress transcription from *scbR* gene. ScbR bound to *O_A_* represses transcription from *scbA* gene, whereas a protein-protein complex, ScbA-ScbR (*AR*) activates transcription from *scbA* gene. Blunt arrow indicates repression and pointed arrow denotes activation. The two mRNAs, *scbR* and *scbA* are shown as *r* and *a*, respectively. Intracellular SCB1 (shown as *C*) is formed from glycerol derivates and β-keto acid by enzymatic action of ScbA (*A*). SCB1 can form a complex with ScbR protein (*CR*), and also diffuse out of the cell (*C_e_*).

Based on genetic and biochemical studies of mutants, Takano and coworkers [21] have shown that ScbR autorepresses its own transcription and that of *scbA*; binding of SCB1 to ScbR relieves the repression of *scbR* and subsequent activation of *scbA* expression enhances SCB1 synthesis. Integrating Takano's original proposed mechanism with recent findings of ScbR repression of CpkO and a probable direct role of ScbA in SCB1 synthesis, a scenario of the regulation of *scbA/scbR* system can be put together (depicted in Figure 1). The molecular mechanism of *scbA* activation has not been elucidated yet. In our model the original hypothesis of the formation of an ScbA-ScbR complex and the activation of *scbA* expression is adopted [20]. *scbA* and *scbR*, located at neighboring positions (*SCO6266* and *SCO6265*, respectively) on the *S. coelicolor* chromosome, are divergently transcribed. ScbR, synthesized at basal levels during early exponential phase, autorepresses its expression by binding to its own promoter region (site *O_R_*), and also represses *scbA* by binding at site *O_A_*. During exponential growth phase, ScbR, which exists mostly in the DNA bound form (no binding of SCB1 or ScbA), suppresses itself and also secondary metabolite biosynthesis by repressing a positive regulator such as CpkO. As the cells progress to transition stage of growth, an increase in SCB1 concentration ensues a corresponding increase in the level of the putative enzyme, ScbA. Upon exceeding a threshold concentration, SCB1 binds to ScbR to form an SCB1-ScbR complex. This binding of SCB1 to ScbR results in the loss of DNA-binding activity of ScbR and a derepression of *scbA* and *scbR*. The interaction of ScbA and ScbR to form an ScbA-ScbR complex is hypothesized to activate transcription of *scbA*. This in turn enhances SCB1 production [19], [20], which reduces the concentration of free ScbR. Consequently, the reduction in free ScbR abolishes the repression of genes in the *cpk* cluster.

The proposed mechanism of *scbA/scbR* regulation is largely consistent with observations reported. However, although a qualitative description can be presented for the time sequence of events of antibiotic synthesis regulation, it is not clear that there exists a realistic combination of parameter values (concentration range of components and binding constants), which enables the hypothesized behavior of the system. Furthermore, as discussed earlier, biologically one expects a good signaling system for antibiotics biosynthesis to have a sharp response to the cue or signaling molecule (in this case SCB1) and to be affected by growth rate. To evaluate the possibility that such a model can indeed provide a robust control of antibiotic synthesis we constructed a mathematical model of the system. The parameter space relevant to physiological conditions and beyond was surveyed to determine the variety of dynamics that are possible with this system.

## Materials and Methods

### Model and simulations

The complete set of equations for the model is listed in Table 1. The binding of repressor protein to the operator sites is at the root of transcriptional regulation, as shown in Equations 1–3. ScbR (*R*) protein, the auto-repressor, prevents its own transcription by binding to the *O_R_* operator site and forming the *O_R_-R* complex as depicted by Equation 1. In addition to binding to the operator site *O_R_*, ScbR protein also forms complexes with ScbA (*A*) protein and intracellular SCB1 (*C_i_*) (Equations 4 and 5). Assuming the binding reaction of *O_R_* is rapid and is essentially in equilibrium, the fraction of unbound operator sites 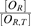 can be derived from Equation 7, where *K_OR_* is the equilibrium dissociation constant 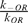. Applying similar assumption of equilibrium, the ratios 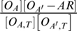 and 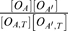 are derived as shown in Equations 8 and 9.

**Table 1:** Equations for ScbA/ScbR Model.

**Rate Equations**	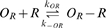 (1)
	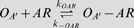 (2)
	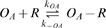 (3)
	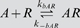 (4)
	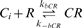 (5)
	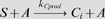 (6)
**Equilibrium Relations**	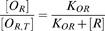 (7)
	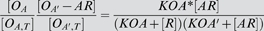 (8)
	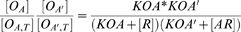 (9)
**Mass Balance Equations**	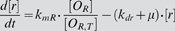 (10)
	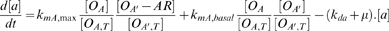 (11)
	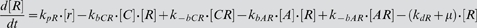 (12)
	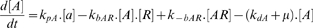 (13)
	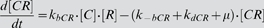 (14)
	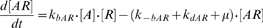 (15)
	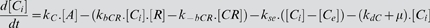 (16)
	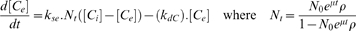 (17)

The transcript of *scbR* (*r*) as shown in Equation 10 is the balance of transcription rate, degradation and dilution due to volume expansion caused by growth. Degradation of *scbR* mRNA is assumed to follow first order kinetics with rate constant *k_dr_*. Transcription rate of *scbR* is considered proportional to the ratio of unoccupied regulatory site to the total regulatory sites, 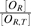. The balance of *scbA* (*a*) transcript (Equation 11) is similar to *r* except that the repressor (*R*) and the activator ScbA-ScbR (*AR*) complex bind to two separated operators, *O_A_* and *O_A′_*, respectively (Equations 2 and 3). *ScbA* transcription rate is maximal when the repressor site, *O_A_*, is unoccupied and the activator site, *O_A′_*, is occupied by the *AR* complex. The first term of Equation 11 depicts the transcription rate as being proportional to the fraction of unoccupied *O_A_* sites and AR bound *O_A′_* sites. In addition, there is a basal rate of transcription when both the sites are unoccupied. The balance of ScbR and ScbA proteins is described similarly (Equations 12 and 13). The synthesis of ScbR protein is modeled as proportional to the *r* concentration. Binding and unbinding of ScbA and ScbR to each other and ScbR to SCB1 also contributes to their balance. However, binding of ScbR to the operator site is neglected since the concentration of operator sites is low. Note that ScbR protein probably exists as a homo-dimer. However, since the functional state of ScbR is always a dimer, the homo-dimer and monomer states do not need to be considered separately. The balance of SCB1-ScbR (*CR*) and ScbA-ScbR (*AR*) complexes is given in Equations 14 and 15, respectively.

The production of the butyrolactone, SCB1, from a glycerol derivative and β-keto acid derivative precursors (*S*) is assumed to be proportional to the concentration of ScbA (first term in the rate Equation 16). For the balance of SCB1 (*C_i_*) (Equation 16) we assume that its transport rate into or out of the cell is proportional to the concentration difference across the cell membrane, as denoted by the *k_se_*(*C_i_*−*C_e_*) term. Degradation of *C_i_* is taken to be a first order process. Equation 17 describes extracellular SCB1 (*C_e_*). The accumulation rate of extracellular SCB1 is affected by cell density. Here *ρ* denotes the volume fraction of a single cell, and *N_0_* is the initial number of cells.

### Growth and transcriptional analysis

*S. coelicolor* M145 (SCP1^−^, SCP2^−^) was used in this study and was maintained as spore suspension prepared from agar plate using R5 medium [22]. Liquid culture was initiated by inoculating ∼10^7^/ml of spores, pregerminated for 8 hr in 2xYT medium, into 50 ml flasks containing 10 ml of modified R5 medium [23]. Stainless steel coils were added to reduce pellet formation and promote dispersed growth. Cultures were maintained at 30°C and 300 rpm in an orbital shaker and cell growth was measured periodically by optical density (OD) at 450 nm. For RNA extraction one-fifth volume of ice-cold stopping solution (5% phenol in ethanol) was added to cell sample to prevent RNA degradation [24]. Cell were harvested by centrifugation at 4°C and stored at −80°C until further use.

Total RNA extraction was performed as described in a previous study [25]. Trace amount of genomic DNA (gDNA) in the RNA samples was digested using Turbo DNA-free™ kit (Ambion, Austion, TX, USA). Reverse transcription was performed using 2 µg of total RNA, 1 µg of random hexamer primer (Amersham Biosciences, Piscataway, NJ) and Superscript III™ (Invitrogen, Carlsbad, CA) according to manufacturer's protocol. RNA was digested by incubation with RNase H (Invitrogen) at 37°C for 20 min. cDNA was stored at −20°C until further use. Real-time quantitative PCR was performed on Mx3000P instrument (Stratagene, La Jolla, CA) using FullVelocity™ SYBR® Green kit QPCR kit (Stratagene). Triplicate reactions were performed and appropriate negative controls were included to confirm the absence of residual gDNA in RNA samples, and primer-dimer formation. The SYBR Green fluorescence was normalized by ROX fluorescence and a threshold of 0.2 was applied on the log(fluorescence) vs. cycle number plot to obtain C_t_ values. The C_t_ value of *SCO5820* (*hrdB*), the major vegetative sigma factor in *S. coelicolor* was used for normalization. The 2^−ΔΔCt^ method [26] was used to compute relative changes in gene expression. The 0 min sample withdrawn just before SCB1 addition was used as the control sample.

Genome-wide temporal transcriptome data for M145 wild-type in a batch liquid culture of modified R5 medium was obtained from a public repository, Gene Expression Omnibus [27] (GEO accession number: GSE8107) [28].

### Steady state and dynamic analysis of mathematical model

To obtain numerical solutions the differential equations were solved using the stiff differential equations solvers ode23s in Matlab®. The fixed points for the equations were computed in Mathematica. Eigenvalues of the Jacobian were used to characterize system stability at a given fixed point. The complete set of kinetic parameters involved in the above model is listed in Table 2. The range of values for each parameter, listed in Table 2, was obtained from the literature [24], [29], [30], [31], [32], [33], [34], [35] The parameter range was explored to determine the capability of such a system to show the desired system dynamics

**Table 2:** Parameter values and their range for which bistability is observed.

Parameter	Description	Range tested	Estimated value for bistability	Range of bistability		Remarks/Reference	Units
				Min. evaluated	Max. evaluated		
***Equilibrium constants***							
*K_OR_*	Binding of ScbR to *O_R_*	10^−5^–10	8.82	5.80	9.50	[33], [34]	nM
*K_OA_*	Binding of ScbR to *O_A_*	10^−5^–10	7.44	7.30	8.30		nM
*K_OA′_*	Binding of ScbA-ScbR to *O_A_*_′_	10^−5^–10	4.35	3.86	4.39		nM
***Other rate constants***							
*k_mR_*	*scbR* mRNA transcription	10^−4^–10	1.8×10^−1^	1.3×10^−1^	2.0×10^−1^	[29]	s^−1^
*k_mA_*	*scbA* mRNA transcription	10^−4^–10	4.5×10^−1^	4.4×10^−1^	5.0×10^−1^		s^−1^
*k_dr_*	*scbR* mRNA degradation	6.6×10^−4^–1.2×10^−2^	1.7×10^−3^	1.5×10^−3^	2.5×10^−3^	[24]	s^−1^
*k_da_*	*scbA* mRNA degradation	6.6×10^−4^–1.2×10^−2^	1.8×10^−3^	1.6×10^−3^	1.9×10^−2^		s^−1^
*k_pR_*	ScbR protein translation	10^−4^–10	3.6×10^−1^	2.5×10^−1^	3.9×10^−1^	[32], [33]	s^−1^
*k_pA_*	ScbA protein translation	10^−4^–10	6.6×10^−2^	6.4×10^−2^	7.3×10^−2^		s^−1^
*k_dR_*	ScbR protein degradation	10^−7^–10^−1^	4.0×10^−3^	2.2×10^−3^	6.7×10^−3^	Half-life of seconds to hours [33]	s^−1^
*k_dA_*	ScbA protein degradation	10^−7^–10^−1^	1.8×10^−3^	1.3×10^−3^	1.8×10^−3^		s^−1^
*μ*	growth rate	0–10^−4^	6.7×10^−5^	0.0	1.0×10^−4^	[35]	s^−1^
*k_C_*	SCB1 synthesis	0–1.7	7.4×10^−1^	6.7×10^−1^	1.7	[30]	s^−1^
*k_dC_*	SCB1 degradation	0–2×10^−4^	6.7×10^−5^	0.0	8.5×10^−3^	Max. half life 1 hr.	s^−1^
*k_bCR_*	Binding of ScbR and SCB1 to form SCB1-ScbR complex	10^−7^–10^−1^	8.3×10^−2^	7.7×10^−2^	1.7×10^−1^	[34]	nM^−1^ s^−1^
*k_−bCR_*	Unbinding of SCB1-ScbR complex	0–10^3^	1.7×10^2^	2.4×10^1^	1.8×10^2^		s^−1^
*k_bAR_*	Binding of ScbA and ScbR to form ScbA-ScbR complex	10^−7^–10^−1^	8.3×10^−2^	8.3×10^−2^	1.2×10^−1^	[34]	nM^−1^ s^−1^
*k_−bAR_*	Unbinding of ScbA-ScbR complex	0–10^3^	6.3×10^2^	4.6×10^2^	6.3×10^2^		s^−1^
*k_se_*	SCB1 secretion	0–4.0×10^−1^	8.3×10^−2^	2.0×10^−2^	9.7×10^−2^	[31]	s^−1^
*k_dCR_*	SCB1-ScbR degradation	10^−7^–10^−1^	6.2×10^−2^	6.1×10^−2^	1.7×10^−1^	[33]	s^−1^
*k_dAR_*	ScbA-ScbR degradation	10^−7^–10^−1^	6.2×10^−2^	5.1×10^−2^	6.9×10^−2^	[33]	s^−1^

## Results

### ScbA/ScbR system as a bistable switch

**Figure 2:**
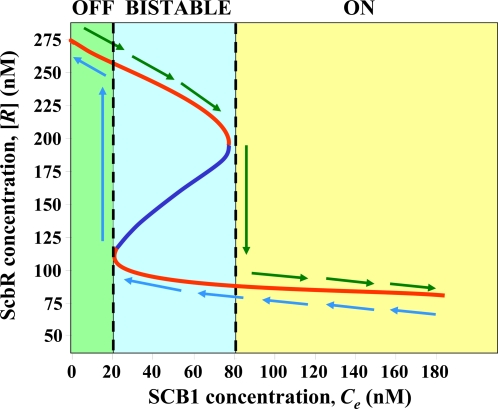
Steady state behavior of ScbA/ScbR system: Steady state ScbR concentration as a function of constant extracellular SCB1 concentration. Red denotes a stable steady state, whereas unstable steady state is depicted by blue. The steady state plot has been divided into three regions: two monostable and one bistable region. The monostable regions are marked as OFF or ON; OFF corresponding to absence of antibiotic and ON corresponding to presence of antibiotic production. Arrows indicate the path from OFF to ON and vice-versa. Hysteresis is indicated by the different paths in either direction (green and light blue arrows).

The bistability of *scbA/scbR* system was evaluated in this investigation. The fixed points of the butyrolactone system in terms of the concentration of ScbR protein ([*R*]) for different extracellular SCB1 concentrations (*C_e_*) are shown in Figure 2. Since ScbR protein is the direct effector of downstream activation of the *cpk* gene cluster, the effect of *C_e_* on ScbR is presented. To determine these fixed points, *C_e_* was kept at a constant level. The stability of each fixed point is denoted by red (stable fixed point) and blue (unstable steady point), respectively. The values of the parameters used in this case along with their source are listed in Table 2. At low concentrations of SCB1 (*C_e_*<21 nM), only steady states with a high [*R*] exist. As *C_e_* increases beyond 21 nM three fixed points of the system emerge until it reaches 76 nM. In two of the regions the steady state is stable (denoted by red in Figure 2) while in the other (blue) region the steady state is unstable. The stable steady states with low free ScbR concentration ([*R*]) correspond to the ON state for expression of genes in *cpk* cluster, while the states with high [*R*] represent an OFF state for expression of *cpk* cluster genes. Cells can exist in either ON or OFF steady states in this range of *C_e_* concentration. At *C_e_*>76 nM, the high [*R*] steady state vanishes and only the ON state exists. This distinction of ON and OFF states by ScbR concentration is in accordance with the mechanism proposed by Takano *et al.* [18]. It is important to note that free ScbR protein exists at primarily two distinct states. It is either greater than 190 nM (OFF) or lower than 110 nM (ON). No intermediate values are admissible for [*R*], and thus its behavior can be approximated as a two-state discrete switch. The steady state profiles of all other species as a function of SCB1 (*C_e_*) concentration are shown in Figure S1.

### Dynamics of Bistable Switch

The steady state behavior of the system is notable for its classical *S*-shaped curve with a region marked by the coexistence of two stable steady states. Although the steady state is marked by a specific level of free ScbR in Figure 2, each steady state is actually characterized by a set of variables (concentration of ScbA protein ([*A*]), transcript of *scbA* (*a*) and *scbR* (*r*)). Cells are initially in an OFF steady state with respect to *cpk* gene expression. When subjected to a step increase to a different level of *C_e_*, cells will exhibit different transient behavior to reach a new steady state. If the concentration of SCB1 is below the threshold concentration of 76 nM, cells will remain in a steady state that is OFF. The ON state, in contrast, is reached only if the *C_e_* concentration is increased beyond the bistable region (*C_e_*>76 nM).

**Figure 3:**
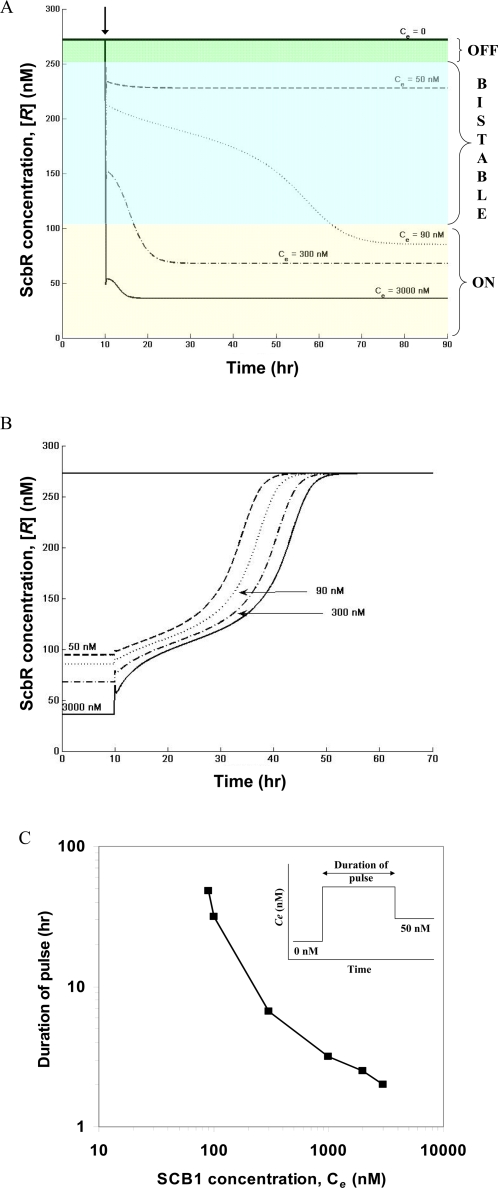
Dynamics of switch between OFF and ON states. (A) Switch from OFF to ON state. Dynamics of ScbR protein in response to a step input of extracellular SCB1 concentration of 50 nM (dashed line), 90 nM (dotted line), 300 nM (dashed dotted line), and 3000 nM (solid line). The step input is applied at 10 hrs as marked by arrow. The system is initially at steady state corresponding to absence of SCB1 in the extracellular medium (*C_e_* = 0 nM). (B) Switch from ON to OFF state. The system is at ON state corresponding to extracellular SCB1 concentration of 50 nM, 90 nM, 300 nM and 3000 nM. At 10 hrs *C_e_* is reduced to zero and the dynamics towards OFF steady state are observed. (C) Minimum duration of step change in SCB1 concentration to switch ON the system. The profile of the step input is shown in the inset. Initially cells are at steady state corresponding to *C_e_* = 0. After exposing to SCB1 at different concentrations for a fixed time, SCB1 concentration is reduced to 50 nM. The minimum duration of the pulse that allows the cells to attain the ON state even when step input is decreased to 50 nM is shown.

The dynamics of such a state-switch upon a step increase in *C_e_* are illustrated in Figure 3a. The system is initially at the high [*R*] steady state corresponding to *C_e_* = 0. A step change of *C_e_* concentration to 50 nM (below switching threshold in the bistable region), 90 nM (slightly higher than switching threshold) and 3000 nM (≫switching threshold), respectively was initiated at 10 hours as indicated in Figure 3a. With a step increase in *C_e_* to 50 nM (Figure 3a), [*R*] decreases from its original steady state value briefly, but stays high in the region of OFF state before it settles to a new, and OFF steady state. At a step input just above the threshold value (90 nM), [*R*] level decreases to the region of ON state gradually. For a step input of 3000 nM, much greater than the threshold concentration, switching happens much faster. The time span it takes to switch to the ON state decreases as *C_e_* increases.

In contrast, for cells which are initially at a ON steady state, a step decrease of concentration causes the state to switch to OFF only if *C_e_* decreases to below the low threshold value of 21 nM (at the minimum bound of bistable region). This is illustrated by the simulation results shown in Figure 3b. Cells initially at an ON steady state, at different external SCB1 concentrations are subjected to a step decrease to *C_e_* = 0. They all evolve to the OFF steady state. It is noted that the time period it takes to switch OFF the system (reaching ScbR concentration of 272 nM, the steady state level of [*R*] at *C_e_* = 0) by removal of *C_e_* is not drastically different for different initial *C_e_* concentrations.

Two characteristics of the bistable system are worth noting. First of all, a sharp shift from the OFF state to the ON state is seen as *C_e_* increases from a low value to reach the threshold concentration of 76 nM. Secondly, hysteresis is evident. The *C_e_* threshold for switching OFF differs from that of switching ON. Cells initially in the ON (low [*R*]) state stay ON even when *C_e_* is decreased below 76 nM until it is reduced to below 21 nM. Accompanying the hysteresis is a range of free ScbR concentrations separating the two regions corresponding to OFF and ON states (shown in blue in Figure 2). In this range of [*R*] the system is unstable, thus neither ON nor OFF. Such an *S*-shaped curve shown in Figure 2 is in contrast to a monotonic curve for which such a region separating ON and OFF states would have been an intermediate state.

The results shown in Figure 3a illustrate that the time period for switching ON is affected by the magnitude of step change in *C_e_*. We hypothesize that SCB1 serves as a signal for synchronization by a voting mechanism. A strong and persistent signal indicates a “majority” response by surrounding population and marks a positive signal and is interpreted as a switch indicator even at a shorter duration. Conversely a weaker signal will be treated as true only if it persists over a longer duration. To corroborate this idea, the duration of exposure to high *C_e_* was varied before it was reduced to 50 nM, a concentration that is below the ON-threshold but is in the bistable region. After *C_e_* was decreased to 50 nM the system may return to OFF state or proceed to the ON state. For each such input profile, the minimum duration of the pulse that is required to switch the system to ON state is determined. Figure 3c shows a plot of this duration with respect to the step input concentration of SCB1. The minimum duration of signal molecule exposure needed for the system to switch decreases sharply from 48 hours at 90 nM to 7 hours at 300 nM and less than 2 hours at 3000 nM.

### Effect of Growth Rate

**Figure 4:**
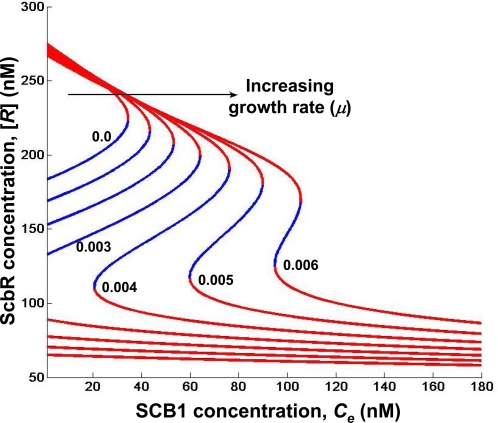
Effect of growth rate on steady states of ScbA/ScbR system. The specific growth rate is varied from 0 to 0.006 min^−1^ in steps of 0.001. For each growth rate, the steady state concentrations are determined for increasing extracellular SCB1 concentrations (*C_e_*). Stable steady states are denoted in red and unstable in blue.

Antibiotics production is generally associated with a decrease in growth rate. In batch cultures SCB1 production also commences upon transition to a slow growth stage [5]. Figure 4 shows simulation results of effect of growth rate (*μ*) on the steady state plot of [*R*] as a function of extracellular SCB1 concentration. The *C_e_* required to switch from an OFF state to the ON state increases with increase in growth rate. It is also noteworthy that the bistable behavior of the butyrolactone system vanishes at very high growth rate. In a fast growing culture, the high threshold concentration of SCB1 required for initiating the downstream response may not be achievable in their native environment.

### Temporal Regulation of the Switch

**Figure 5:**
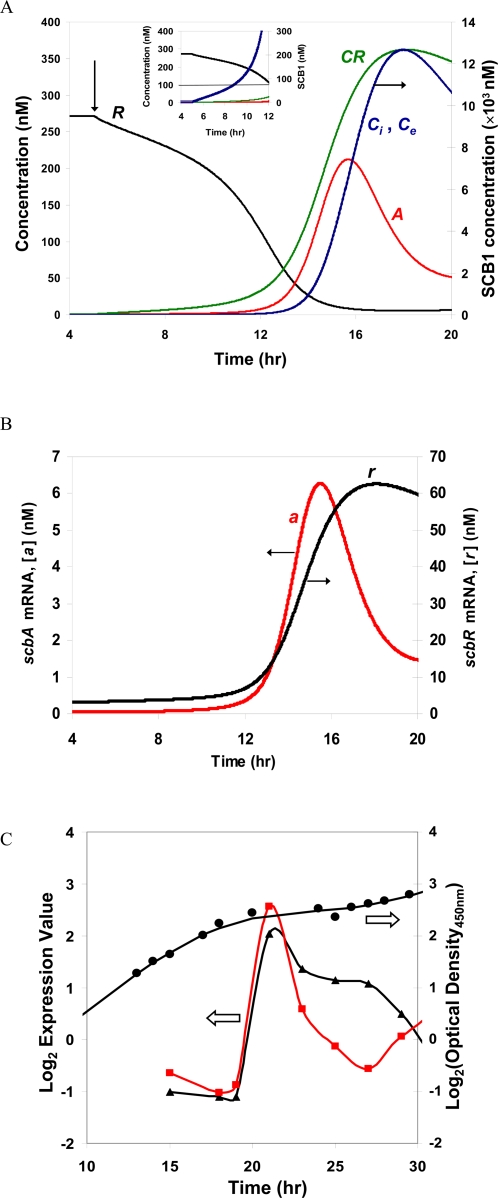
Dynamics of system in response to extracellular accumulation of SCB1. Arrow indicates the time when accumulation of SCB1 was allowed. The dynamics of *C_e_* was modeled as Equation 17 in Table 1. (A) Concentration profiles of ScbR (*R*) (black), ScbA (*A*) (red), SCB1-ScbR complex (*CR*) (green), intracellular and extracellular SCB1 (*C_i_*, *C_e_*) (blue). (B) mRNA profiles of *scbA* (*a*) (red) and *scbR* (*r*) (black). *ρ* = 0.001 was used for this simulation. (C) Transcript profiles of *scbA* (*a*) (red square) and *scbR* (*r*) (black triangle) as measured from microarray experiments. The growth curve is also shown (black circle).

In its natural habitat, *S. coelicolor* responds to the presence of the signaling molecule SCB1 produced by itself and neighboring cells. The rate of increase of SCB1 is intricate, being affected by the density of cells as well as the number of responders. In any case, the change in signaling molecule concentration is more likely to be a ramping function than a step increase. The dynamics under such a gradient rise of *C_e_* are illustrated in Figure 5. Initially cells are in the OFF state in the absence of any SCB1 (*C_e_* = 0 nM). The increase in SCB1 is simulated by a very low rate of intracellular SCB1 (*C_i_*) synthesis, which is afforded by the basal level of ScbA. This SCB1 diffuses from the cells into an extracellular sink. At 5 hrs (indicated by arrow), SCB1 produced intracellularly is allowed to diffuse and accumulate in the extracellular environment. This is simulated by including Equation 17 in the model. As extracellular concentration of SCB1 increases, the intracellular level also increases. The increase in *C_i_* also leads to an increase in the concentration of SCB1-ScbR (*CR*) complex, triggering a decrease in the level of of free *R* (Figure 5a). Repression by *R* is relieved by this decrease in free *R*, and an increase in the concentration of *scbA* and *scbR* transcripts is observed (Figure 5b) followed by an increase in the corresponding protein concentrations. Initial build-up of *C_e_* is slow. However, as *C_e_* rises above the threshold of 76 nM, ScbA begins to accumulate rapidly (Figure 5a) and *C_e_* and *C_i_* levels increase rapidly (see change in slope of *C_e_* in Figure 5a inset). The system switches to the ON state. The transient behavior of the free form repressor protein, *R*, and the SCB1-bound form, *CR* are shown in Figure 5a. Note that the while the level of free *R* decreases, the total concentration of *R* protein (both free and SCB1-bound forms) actually increases in the ON state.

### Comparison with Experimental Data

The consistency of the simulation results of our model with experimental observation was examined. Figure 5c illustrates that under conditions when *C_e_* rises during transition phase, the mRNA levels of both *scbA* and *scbR*, measured by DNA microarrays, increase transiently to reach a peak before subsiding to a lower level. The simulated results of *scbA* (*a*) and *scbR* (*r*) transcript (Figure 5b) closely resemble their time profiles observed experimentally (Figure 5c). The duration of the transient surge of *a* is somewhat shorter than that of *r* as seen experimentally. The model does not accurately capture the decrease in *r* levels. This could potentially be due to the presence of another factor involved in degrading the transcript in stationary phase. The experimental profiles of *r* in a *scbR* deletion mutant (M752) [21] support such a hypothesis.

To further validate the model, a step increase in SCB1 concentration in an early stage culture prior to *scbA* expression, was carried out and the results were compared to model prediction. To five exponentially growing (OD_450_∼0.8) cultures of wild-type (M145), SCB1 was added at concentrations of 0, 5, 25, 50, and 300 nM, respectively. Thereafter samples were taken at 0, 30 and 120 min and probed for transcript levels of *scbA*, *scbR*, and gene *cpkI* (*SCO6282*). *cpkI* is located in the *cpk* cluster whose expression is directly controlled by the pathway-specific activator, CpkO [18]. Thus, *cpkI* transcript serves as an indicator of the downstream effect of the switch.

**Figure 6:**
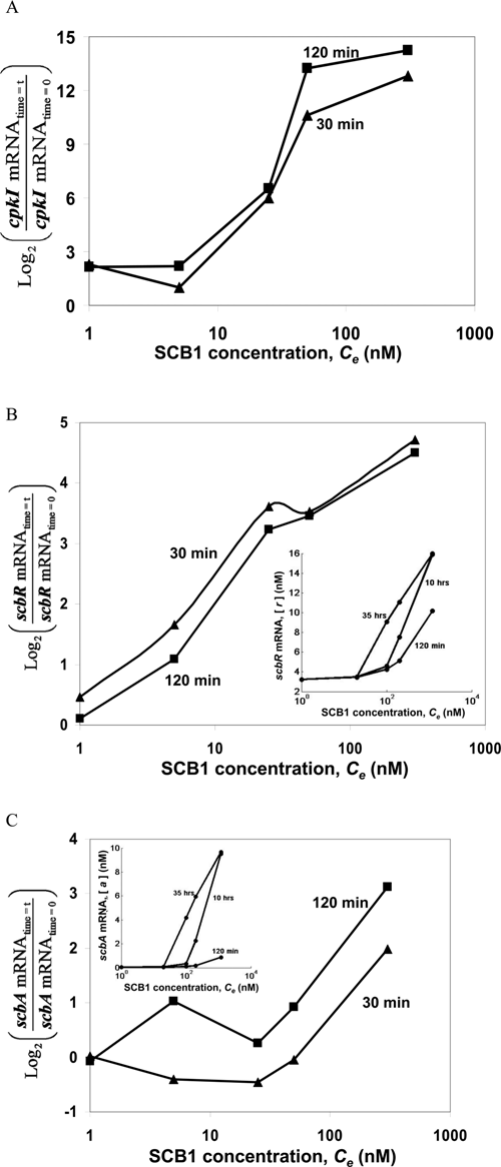
Perturbation of system by exogenous addition of SCB1 at different concentrations. Comparison of experimental results with simulations. SCB1 was added at 0, 5, 25, 50 and 300 nM to exponentially growing liquid cultures of M145 at OD_450_∼0.8. The transcript levels of (A) *cpkI*, (B) *scbR* and (C) *scbA* were measured at 30 min (triangles) and 120 min (squares) after addition of SCB1, using real-time quantitative PCR. The threshold cycle (*C_t_*) values at 30 and 120 min were normalized with that *C_t_* values at 0 min (SCB1 = 0 nM). Simulation results at various time points in response to SCB1 step input of 0, 20, 100, 200 and 1200 nM, are shown in inset of panel (A) ScbR protein, (B) *scbR* transcript and (C) *scbA* transcript.

*cpkI* transcript levels observed at two different time points after addition of SCB1 at different concentrations are shown in Figure 6a. At high SCB1 concentrations (*C_e_* = 50 and 300 nM), *cpkI* transcript level was two to four orders of magnitude higher than that observed low SCB1 concentrations (*C_e_* = 0 and 5 nM). Transcripts of both *scbR* and *scbA* show a similar increasing trend with increasing SCB1 concentration (Figure 6b and c), although the difference between high and low SCB1 concentrations is not as pronounced as the gene *cpkI* that they regulate. It is also interesting to note that SCB1 addition has a more pronounced effect on *scbR* transcript compared to *scbA* transcript, particularly at low SCB1 concentrations (<100 nM). The simulation results predicting similar trends for both *scbR* and *scbA* are shown in insets in Figure 6b and c, respectively.

## Discussion

Several important physiological processes operate in discrete states [36], [37], [38]. Notable examples include sporulation vs. vegetative growth for bacteria, differentiation vs. self-renewal state of stem cells, and lysogeny vs. lytic state for bacteriophage lambda. At any given time, a cell can only exist in either of the two states. This is in contrast to the vast majority of physiological processes where, in addition to a “high” activity state and a “low” activity state, a continuum of intermediate states exists between the two extremes [39]. In contrast to this graded response, the transition from one state to the other in the discrete system is discontinuous like a switch, going from one to the other without a true intermediate state.

The discrete states arise as a consequence of the genetic and biochemical network responding to environmental and developmental signals. The binary states can often be represented in terms of the concentration of a regulatory effector, in response to stimuli. Once the level of the effector reaches a certain threshold level, the subsequent reactions switch the cellular process from one phenotype to the other.

The physiological states of secondary metabolite synthesis, including antibiotics production, and vegetative rapid growth are traditionally thought of as disjunct states; secondary metabolite production is suppressed in rapidly growing cells until growth ceases or at least drastically slows down. On agar plates, secondary metabolism often coincides with sporulation for bacteria such as *S. coelicolor*. The transition from vegetative growth (OFF state) to secondary metabolite production (ON state) is likely to behave like a switch, as is sporulation. Due to the toxic nature of some secondary metabolites, such as antibiotics, it is essential that the ON state be synchronized among individuals in the community. γ-butyrolactones potentially act as quorum sensing type of molecules in *Streptomyces* spp. to regulate the onset of secondary metabolite synthesis. We have examined the regulation of γ-butyrolactone SCB1 and the expression of genes in cryptic type I polyketide cluster in *S. coelicolor* by the gene pair *scbA* and *scbR*. In response to an increase in concentration of signaling molecule, SCB1, the free repressor protein (*R*) transits from a high concentration that represents OFF state to a low concentration that corresponds to ON state.

A switch-like response to signaling molecule concentration can be achieved either by ultrasensitivity or bistability in the relationship between regulatory repressor protein and the signaling molecule. In the former case, the two states are separated by a threshold value of the signaling molecule; on one side of the threshold the system is in the OFF state, on the other it is in the ON state. In the latter case the ON and OFF states reside in two distinct regions of high and low concentration of the regulatory protein (*R*), respectively. Between the two monostable states exists a bistable region in which the system can be in either the ON state or the OFF state depending on the initial condition. In the first scenario, the case of ultrasensitivity, a tiny difference in (*R_crit_*, *C_crit_*) separates the two states; upon crossing the critical point the systems switches from one state to another. Such a system will require every component involved to be highly fine-tuned with little leeway for fluctuations. In contrast, a bistable system exhibits hysteresis; the path of switching ON from an OFF state is different from that of switching OFF from an ON state. With a bistable system, both the effector (*R*) and signaling molecule (*C*) concentrations for ON and OFF states are well separated, giving rise to the switch behavior where the ON and OFF states are separated by a region of bistability. The simulation results demonstrate that the switch to an ON state from OFF requires an exposure to concentrations beyond the bistable region. Furthermore, as the growth rate increases the concentration of signaling molecule required to switch ON increases (Figure 4). At very high growth rate the bistable behavior disappears (data not shown). The simulated dynamic behavior of *scbA* and *scbR* in a liquid culture resembles that of experimental observation.

Consistent with the voting hypothesis, i.e. a strong and fast rise in signaling molecule concentration is indicative of a positive signal as opposed to noise, the minimum duration of exposure to signaling molecule decreases with increasing concentration (Figure 3c). The bistable plot of [*R*] vs. *C_e_* shown in Figure 2 illustrates that, for a system initially at an OFF state, as SCB1 concentration increases it will cross over to an ON state at *C_e_* = 76 nM. The experimental results shown in Figure 6 are qualitatively consistently with model predictions. Since the organism triggers secondary metabolite synthesis at high cell concentrations as cells enter a late transition stage, the exogenous SCB1 addition experiment was carried out in early stage of the culture. The addition of SCB1 to exponentially growing cultures induced transcription of a gene encoding a putative reductase enzyme in the *cpk* cluster. The induction was indeed concentration-dependent and no other exogenous factors were needed. Although there is no experimental evidence on quantitative expression levels of the repressor protein *R* during the culture period, a 2D-gel-based proteomics study identified *R* protein from cultures harvested in the transition stage [40] (http://dbkgroup.org/s_coeli/referencegel/index.php). The growth rate of *S. coelicolor* at the time of SCB1 addition was substantially higher than that used for simulation in Figure 2. That may have contributed to the difference in numerical values between experimental observation and simulation results. The bistability shown in Figure 2 also implies that for a system originally in an ON state, reducing *C_e_* should increase the concentration of free ScbR protein gradually until *C_e_* reaches below 21 nM. At this point, the switch should turn OFF accompanied by a sharp increase in *R*. However, the reversibility of a bistable system is not assured, as once the system is turned ON, additional biological processes may become active to prevent the reversal via the original route.

Recognizing the importance of bistability in providing a robust switch system, we searched the parameter space within the constraints of literature values of parameters and identified a range in which bistability of *R* in terms of *C_e_* is realizable. The value of each parameter was varied individually over a range while keeping all the other parameters constant at the nominal value listed in Table 2. For each new parameter value, the steady state response to varying levels of external inducer concentration, *C_e_* was determined. Table 2 summarizes the range of values for each parameter within which bistability is observed. Detailed plots for each parameter are shown in Figure S2. The system exhibits a region of bistability within at least 40% variation in the value for all but five of the parameters. All five of the most sensitive parameters are involved in modulating the concentration of ScbA protein: transcription and translation, mRNA and protein degradation, and binding of *A* to *R* to form the *AR* complex. The bistability behavior appears to be dependent on a delicate balance of these reactions to maintain the level of ScbA protein in a range. Excess of ScbA protein can lead to hyperproduction of SCB1 causing the cells to always exist in the ON state. This is reflected by a decrease in the switching threshold from OFF to ON state as *k_mA_* or *k_pA_* are increased. Similarly, as *K_OA_* increases, the increasing repression of *scbA* transcript by *R* keeps the cells at the OFF state.

Although the system may be sensitive to individual parameter variations, it is robust in the sense that bistability can be exhibited over a wide range of parameter values. Individual parameters can be varied over a much wider range when two parameters are varied in combination. For example when parameters *k_mR_* and *k_dr_* are varied individually less than 30% variation is allowed. However, when varied in combination such that the ratio of the two parameters is unchanged, five-fold variation in each parameter still yields similar results (See Figure S3 for various such combinations of parameters).

Bistability as a possible mechanism of realizing the switch behavior in biological systems has been discussed previously for lambda phage lysis-lysogeny [41], the sinI-sinR and sigmaF networks in *Bacillus subtilis* [32], [42], the *lac* operon in *E. coli* [43], [44] and for the sonic hedgehog (Shh) network that controls a number of critical cellular developmental decisions between distinct fates in vertebrates [45]. In these studies, as well as in the current study, a classical reaction engineering approach was taken to demonstrate that the equations describing the system indeed give rise to a behavior where two regions of monostable steady states are separated by a segment of multiple steady states, as originally depicted for continuous stirred tank reactors by Aris and Amundson five decades ago [46], [47]. It should be noted that the term bistability is also used in a different context in describing the experimental observation of different developmental patterns coexisting in a population [38], [48], such as the subpopulation of *B. subtilis* existing in competence state in a stationary culture [49]. These phenomena of coexisting stable subpopulations may have much in common with the bistable behavior illustrated mathematically and reported in this and other studies; however, whether all of them exhibit multiple steady states is yet to be examined.

There has been discussion on the general architecture of a genetic network that would give rise to a switch-like behavior [50]). A two-gene repressor system [51], [52], where each of the two genes negatively regulates the synthesis of the other; co-operativity, where binding of one molecule improves the binding of second molecule [51]; and positive feedback [53] have all been shown to potentially enable bistability. The *scbA/scbR* system in *S. coelicolor* has both positive feedback and negative feedback loops. The OFF steady state is characterized by high levels of free ScbR protein and basal levels of ScbA protein. In the ON steady state, ScbA is present at greater levels, leading to self-activation and subsequent increase in SCB1 production. (The binding of SCB1 and ScbA to ScbR further suppresses its repressor activity.) The dominant interactions in each of the steady states are shown in Figure S4.

Although the working model of *scbA/scbR* system hypothesized the formation of ScbA-ScbR complex that activates SCB1 synthesis, no experimental evidence for an interaction between ScbA and ScbR proteins has been reported. Also, recent studies strongly suggest an enzymatic role of ScbA in SCB1 synthesis. We formulated a simplified model, where ScbA protein does not form a complex with ScbR, but instead activates its own transcription by binding to its operator region (Text S1). The existence of three fixed points was demonstrated analytically for this simplified system. Further, the fixed points of the system for this model were determined for a set of parameter values and their stability was examined. The plot of [*R*] as a function of *C_e_* (Figure S5) bears many similarities with the bistable plot of the working model (Figure 2). The system exhibits hysteretic bistability–free ScbR protein exists primarily in two different steady state zones corresponding to ON and OFF state of expression of *cpk* cluster genes. Thus, the formation of ScbA-ScbR complex is not a strict requirement for bistable behavior of this system. However, it is important to note that a direct or indirect positive feedback of ScbA is essential for the switch-like behavior. Interestingly, evidence for the role of ScbA in self-activation has been reported previously [21].

γ-butyrolactones are widespread in *Streptomyces* species and are shown to be important in the regulation of antibiotic production. The gene network comprising two-genes and the γ-butyrolactone signaling molecule as the central element of the onset of antibiotic biosynthesis also appears to be common among those species investigated. Some features of this genetic network are conserved such as the loss of DNA-binding activity of repressor protein upon forming a complex with the signaling molecule. However, the dynamic behavior of this two-gene system varies widely in different *Streptomyces* species. The transient rise and fall of *scbA* and *scbR* transcript levels seen in *S. coelicolor* is not observed in all *Streptomyces* species. In *S. virginaie* the expression of *barA* and *barB* (the homologues of *scbA* and *scbR*, respectively) rises monotonically, coinciding with a sharp increase in butyrolactone concentration [6], [54] and turning ON the production of the antibiotic virginiamycin. It is conceivable that the *barA/barB* network also exhibits bistable behavior and acts as a switch, although this has not been shown. At least it is clear that the basic two-gene framework of *scbA* and *scbR*, with one autorepressor and one amplifier acting via production of the signaling molecule, is rather versatile and capable of producing different dynamic behaviors. Using the model we developed and expanding the parameter value from that reported in this study, it can be shown that the system is indeed capable of generating many different kinds of dynamics in addition to bistability, including graded, pulsed or oscillatory responses to extracellular butyrolactone, which may all result from responses of the bacteria to multiple environmental situations. The present model could form a basis for deciphering the ubiquitous butyrolactone system in other *Streptomyces* species. In addition, similar models can be envisaged for several signaling molecule-based systems found in many bacteria, which have been shown to play an increasingly important regulatory role in protecting the organism against a harsh environment, or for pathogenicity, or for inter- and intra-species communication.

## Supporting Information

Figure S1 Steady state concentration of species in the ScbA/ScbR network for constant extracellular SCB1. The stable and unstable steady states are represented by red and blue, respectively. The steady state concentrations of (A) *scbR* mRNA (*r*), (B) scbA mRNA (*a*), (C) ScbA protein, A, (D) SCB1-ScbR complex, (CR), (E) ScbA-ScbR complex (AR); (F) intracellular SCB1 (*Ci*) are plotted.
(0.22 MB PDF)

Figure S2 Effect of parameter perturbation on steady state response of butyrolactone system to constant extracellular SCB1. Each plot shows the results of simulation in which only one parameter is varied, keeping the rest constant at the nominal values listed in Table 2. The values of the parameter being varied are shown next to the respective steady state plot. The dashed line in each plot corresponds to the nominal parameter values. The parameter being varied is (A) K_OR_, (B) K_OA_, (C) K_OA′_, (D) k_mR_ (E) k_mA_ (F) k_dr_ (G) k_da_ (H) k_pR_ (I) k_pA_ (J) k_dR_ (K) k_dA_ (L) k_C_ (M) k_dC_ (N) k_bAR_ (O) k_−bAR_ (P) k_bCR_ (Q) k_−bCR_ (R) k_dCR_ (S) k_dAR_ (T) k_se_
(0.15 MB PDF)

Figure S3 Effect of two parameter perturbation. For each simulation, two parameters are varied such that their ratio is constant. Dotted line corresponds to the nominal parameter values in Table 2. The two parameter combinations are (A) k_mR_/k_dr_, ratio of transcriptional rate constant to degradation rate constant of *scbR* mRNA. (B) k_mA_/k_da_, ratio of transcriptional rate constant to degradation rate constant of *scbA* mRNA, (C) k_pR_/k_dR_, ratio of translational rate constant to degradation rate constant of ScbR protein, (D) K_OA_/k_mA_, ratio of equilibrium binding constant of ScbR to O_A_ operator to transcriptional rate constant of *scbA* mRNA and (E) k_bCR_/k_dCR_, ratio of rate constant for dissociation of CR complex to degradation rate constant of CR complex.
(0.53 MB PDF)

Figure S4 Physical description of the two steady states. (A) OFF state corresponding to high [R] or low [A]; (B) ON state corresponding to the low [R]. The solid line represents the dominant reactions in each steady state. Dashed lines show otherwise.
(0.02 MB PDF)

Figure S5 Steady state behavior of simplified *ScbA*/*ScbR* system. The steady state concentration of ScbR protein, [R], is plotted as a function of extracellular SCB1 concentration, Ce. (A) Steady state plot when basal transcription rate of *scbA* is neglected. (B) Steady state plot when *scbA* is transcribed at a basal rate. Stable steady states are denoted in red, while unstable steady states are marked in blue. Open circles denote the steady state corresponding to [A] = 0, whereas filled circles denote the steady states corresponding to [A]≠0. The transition point beyond which the fixed state corresponding to [A] = 0 becomes unstable is marked by an arrow. At this point, one of the fixed roots corresponding to also disappears.
(0.04 MB PDF)

Text S1 Analytical steady state analysis for a simplified ScbA/ScbR system
(0.14 MB DOC)
