# Supplementary figures and images for: A Bistable Gene Switch for Antibiotic Biosynthesis: The Butyrolactone Regulon in Streptomyces coelicolor
Source: PLoS One. 2008 Jul 16;3(7):e2724. doi: 10.1371/journal.pone.0002724 (PMC2444045; doi:10.1371/journal.pone.0002724)

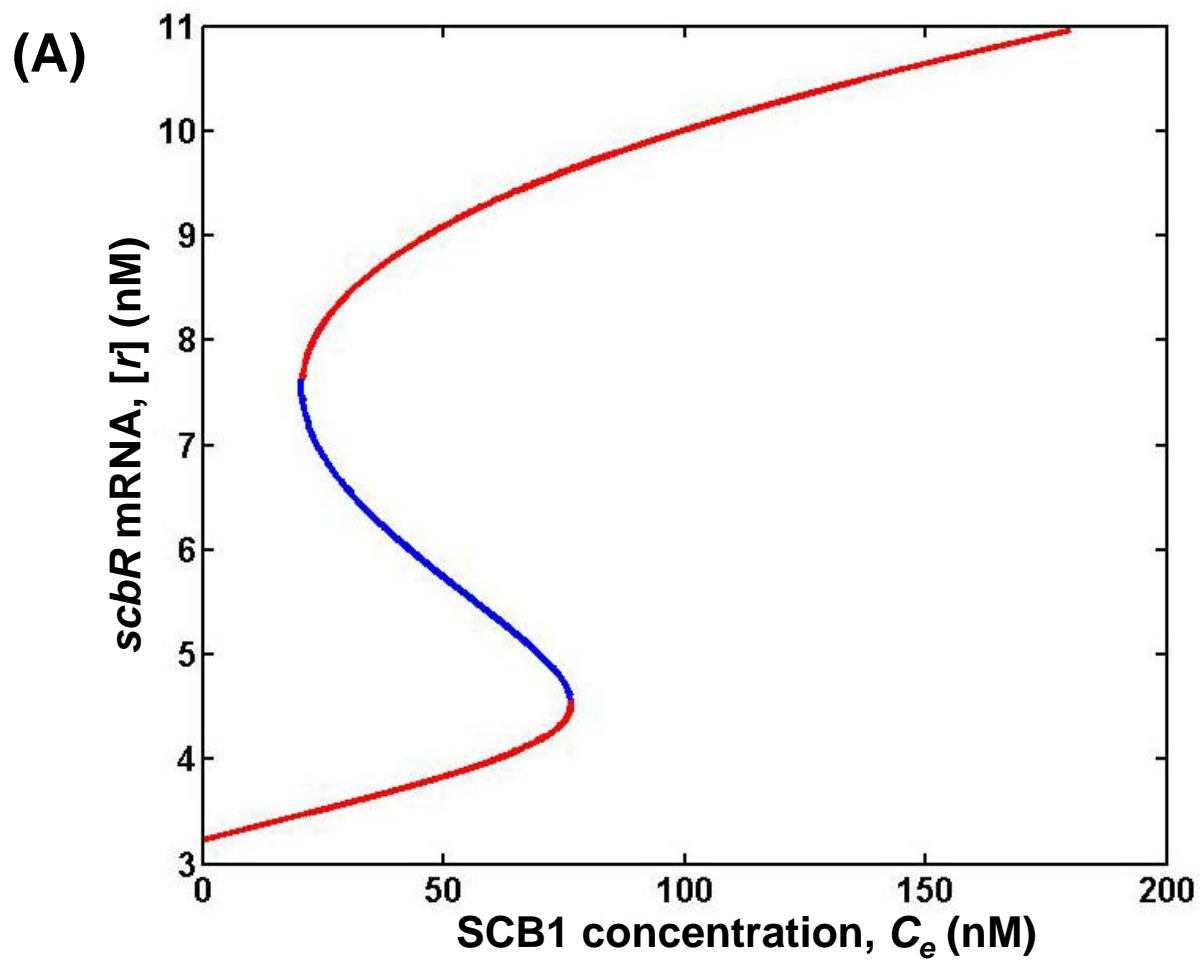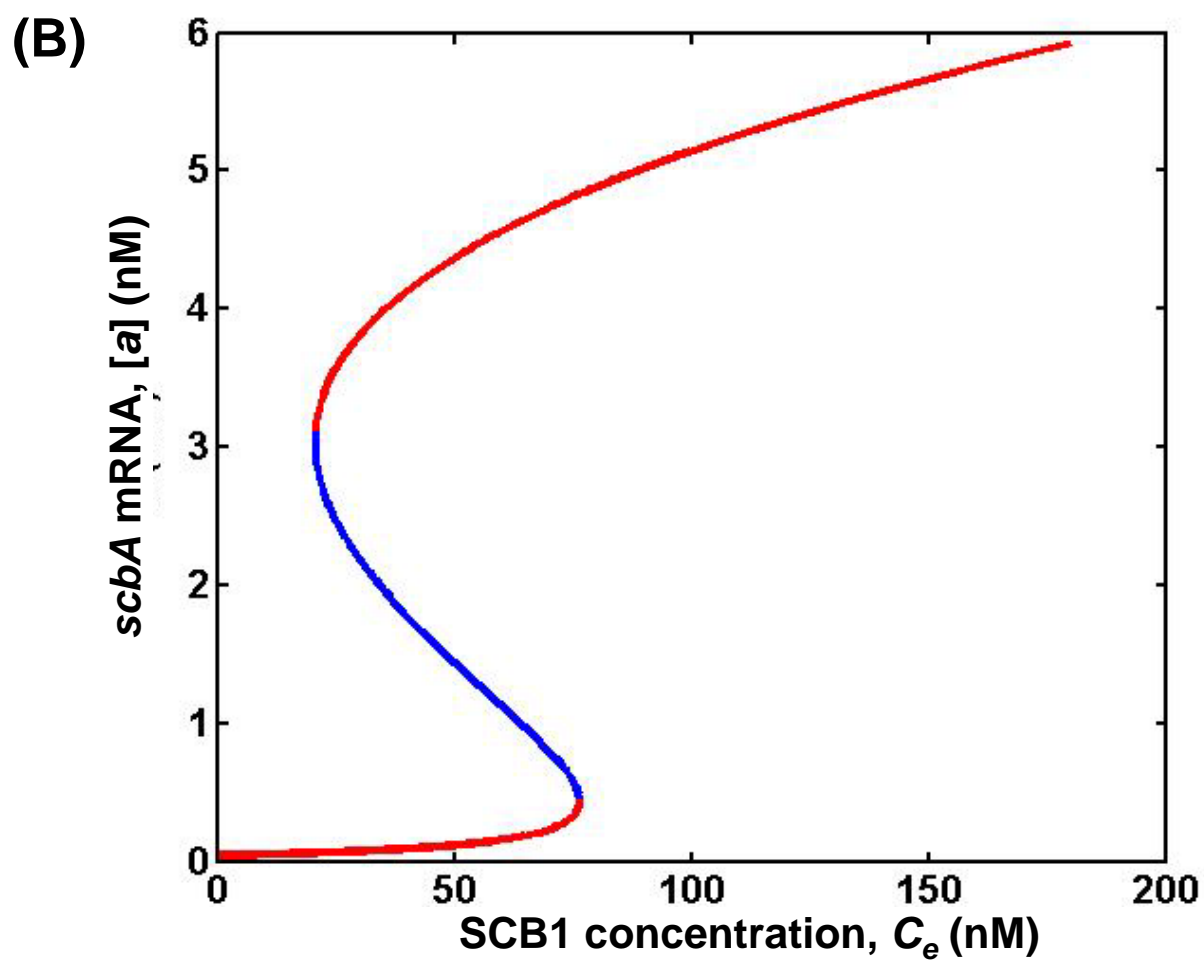

(C)

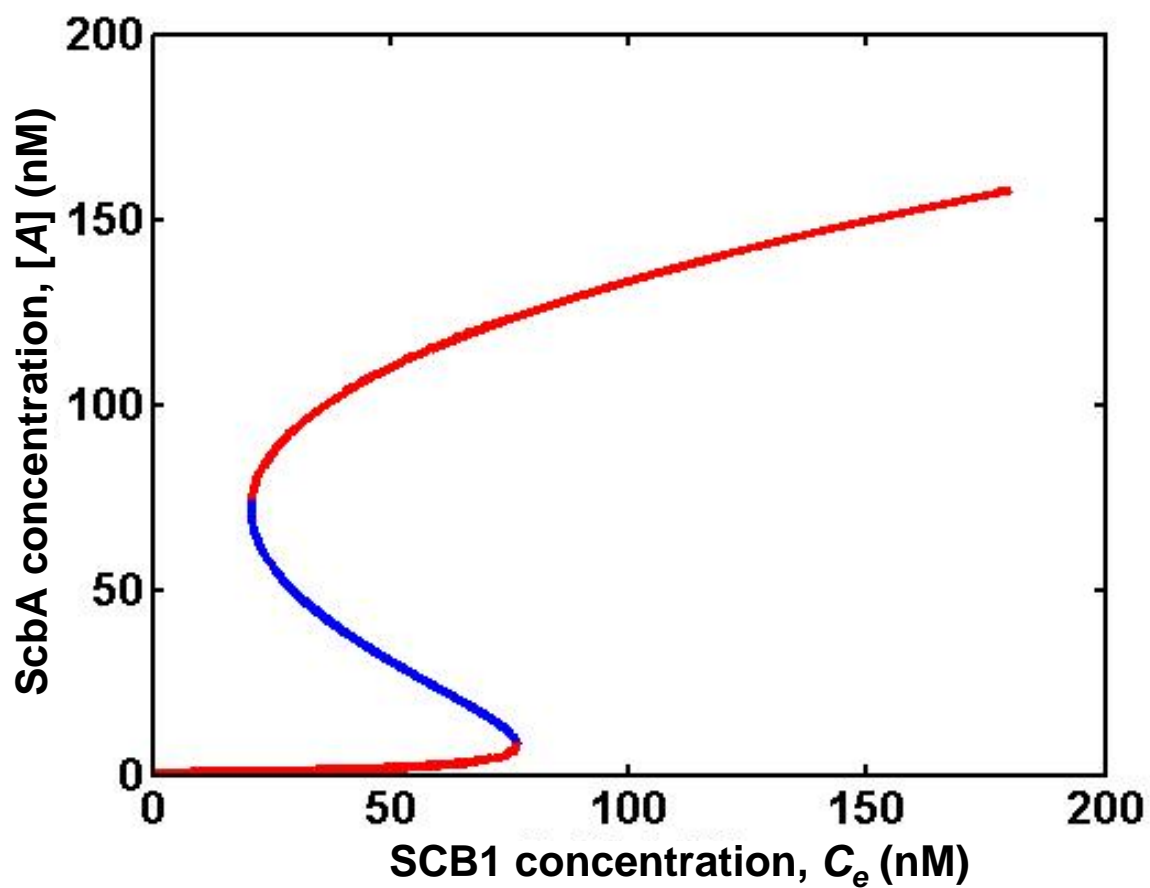

(D)

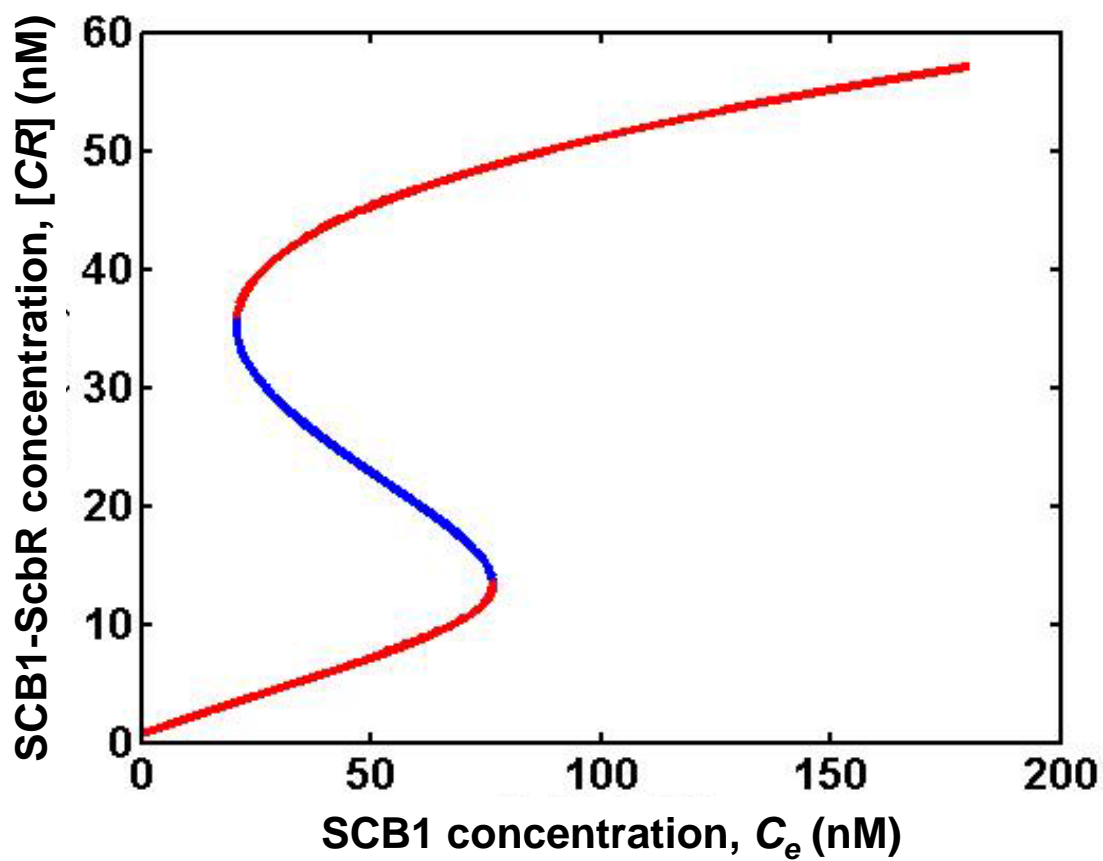

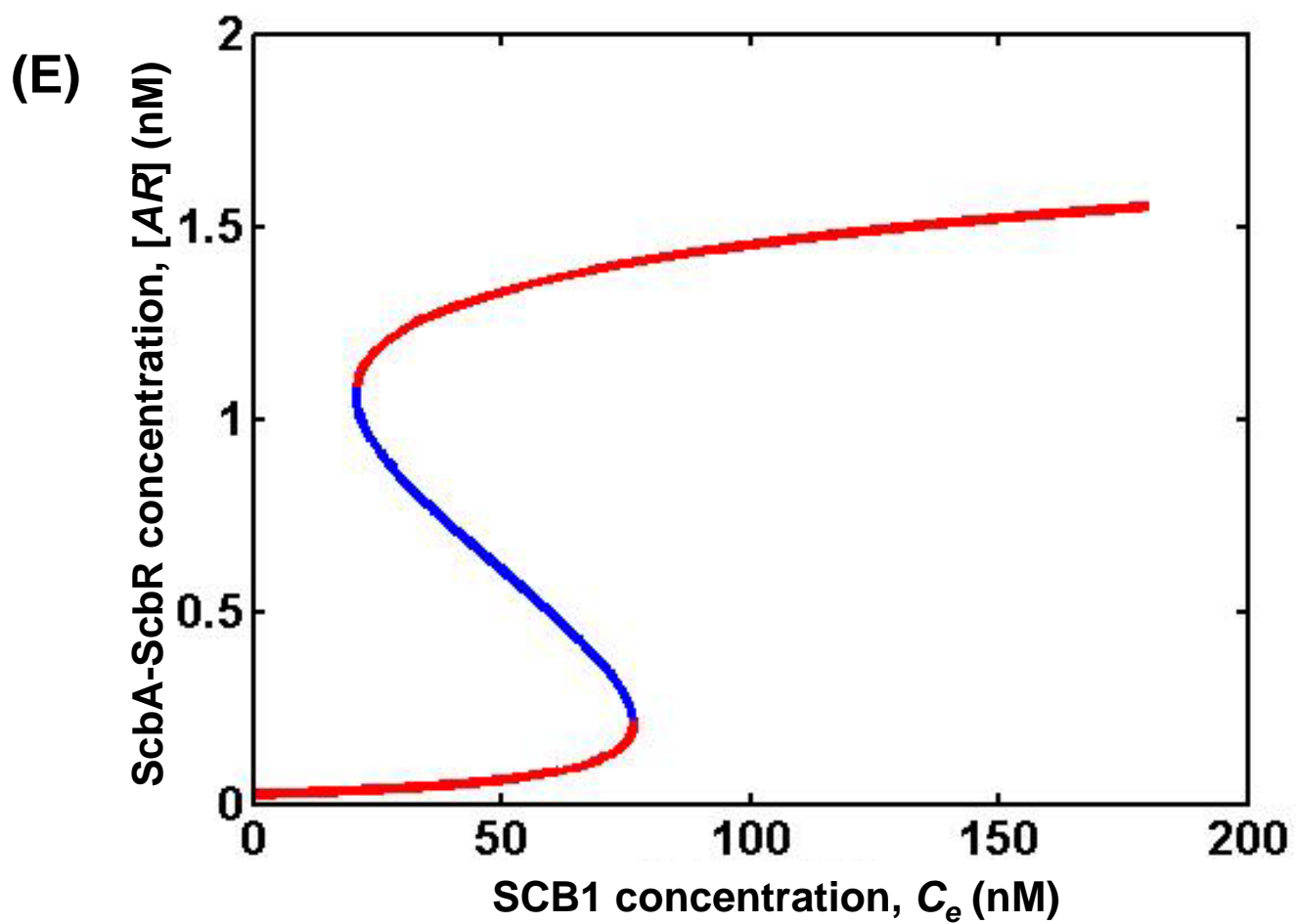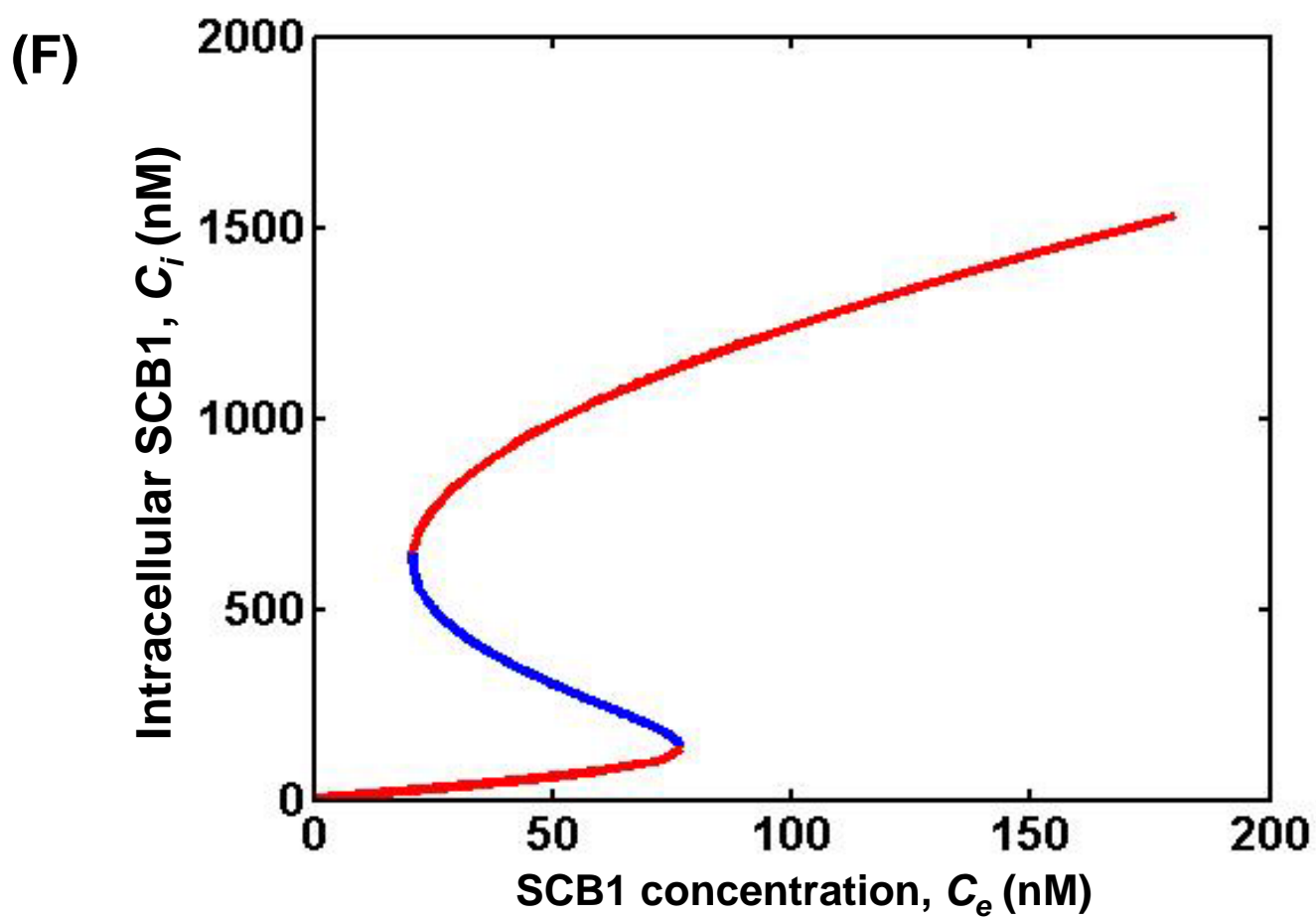

Supplement: Figure S1 — Steady state concentration of species in the ScbA/ScbR network for constant extracellular SCB1. The stable and unstable steady states are represented by red and blue, respectively. The steady state concentrations of (A) scbR mRNA (r), (B) scbA mRNA (a), (C) ScbA protein, A, (D) SCB1-ScbR complex, (CR), (E) ScbA-ScbR complex (AR); (F) intracellular SCB1 (Ci) are plotted. (0.22 MB PDF) [file pone.0002724.s001.pdf]

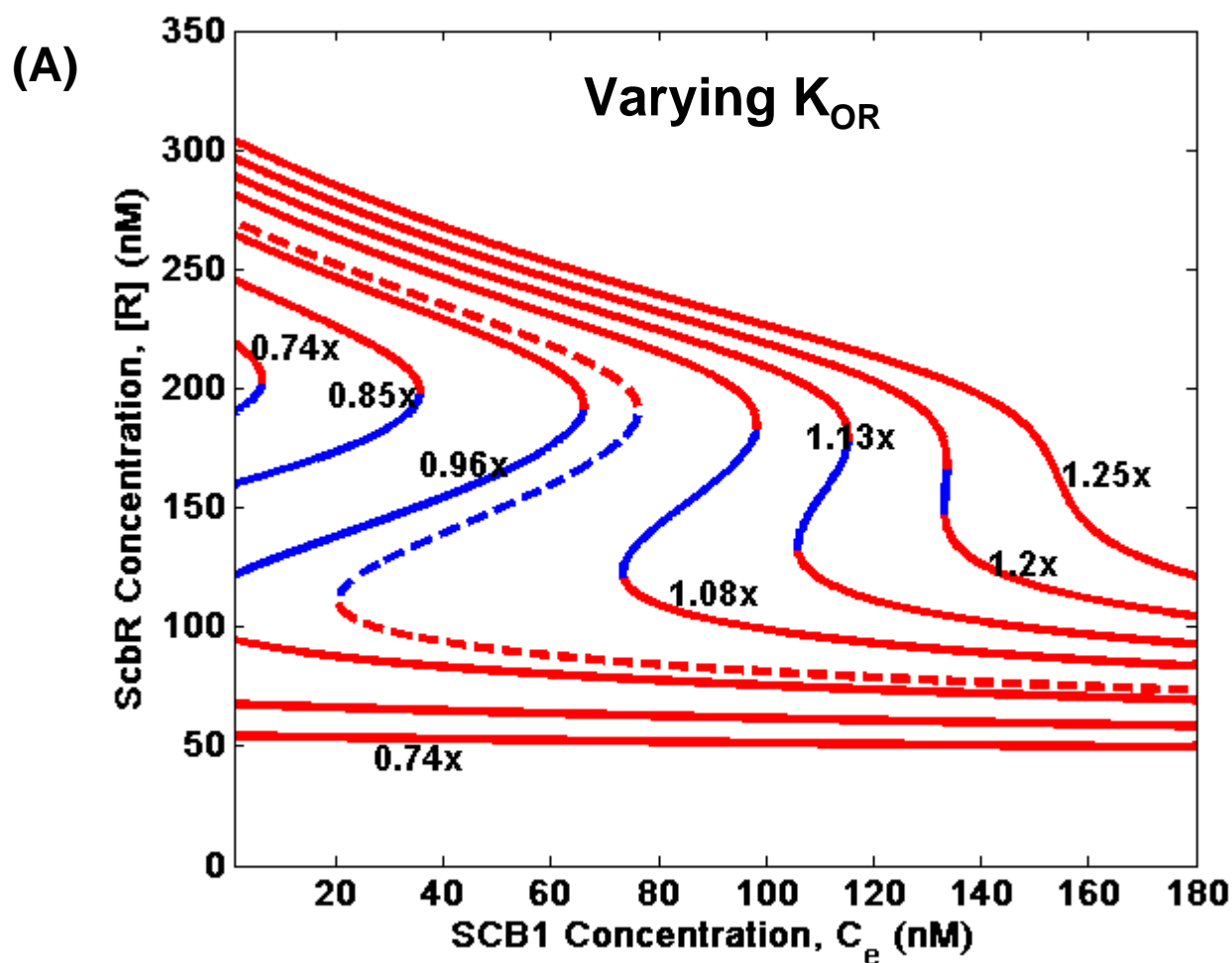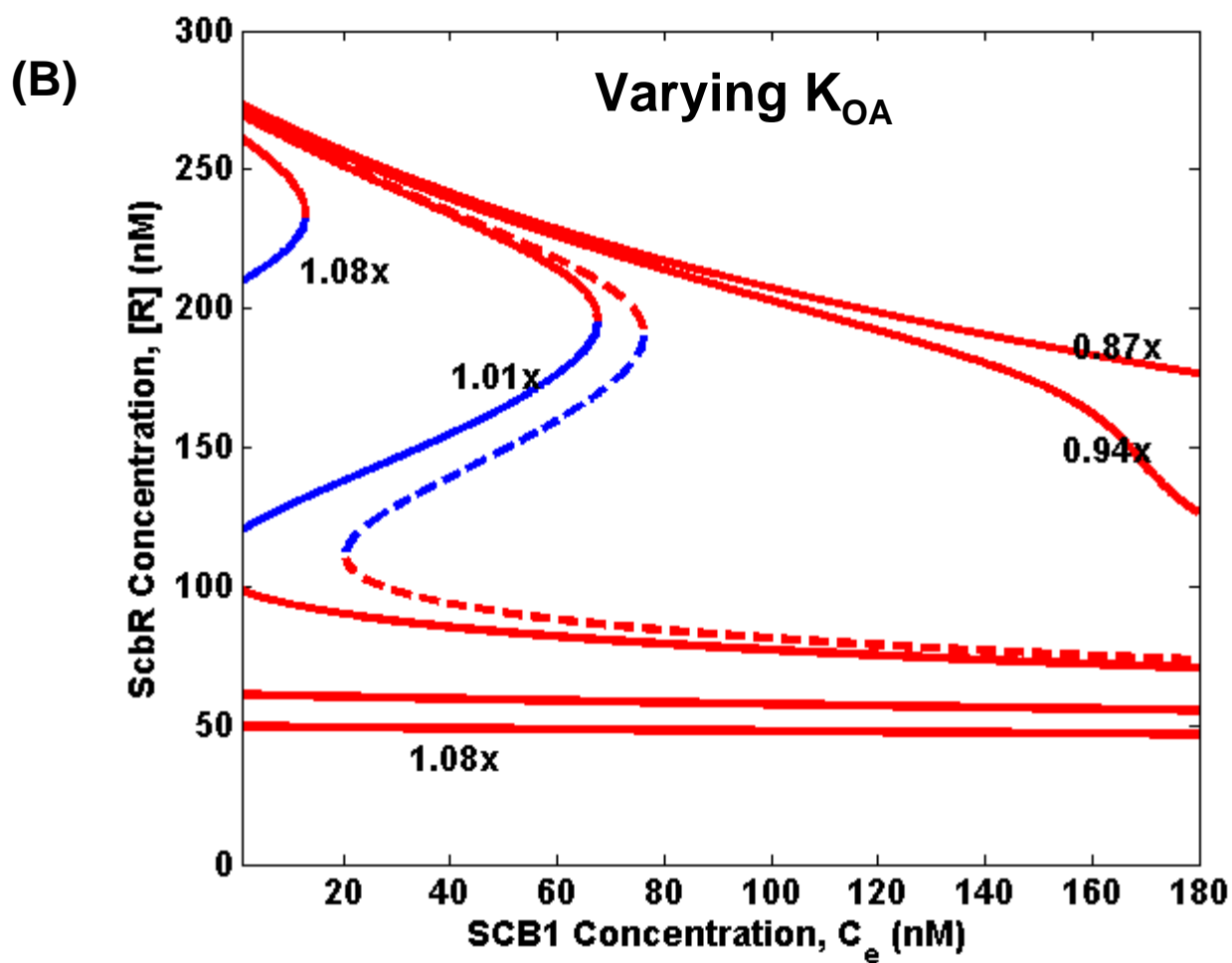

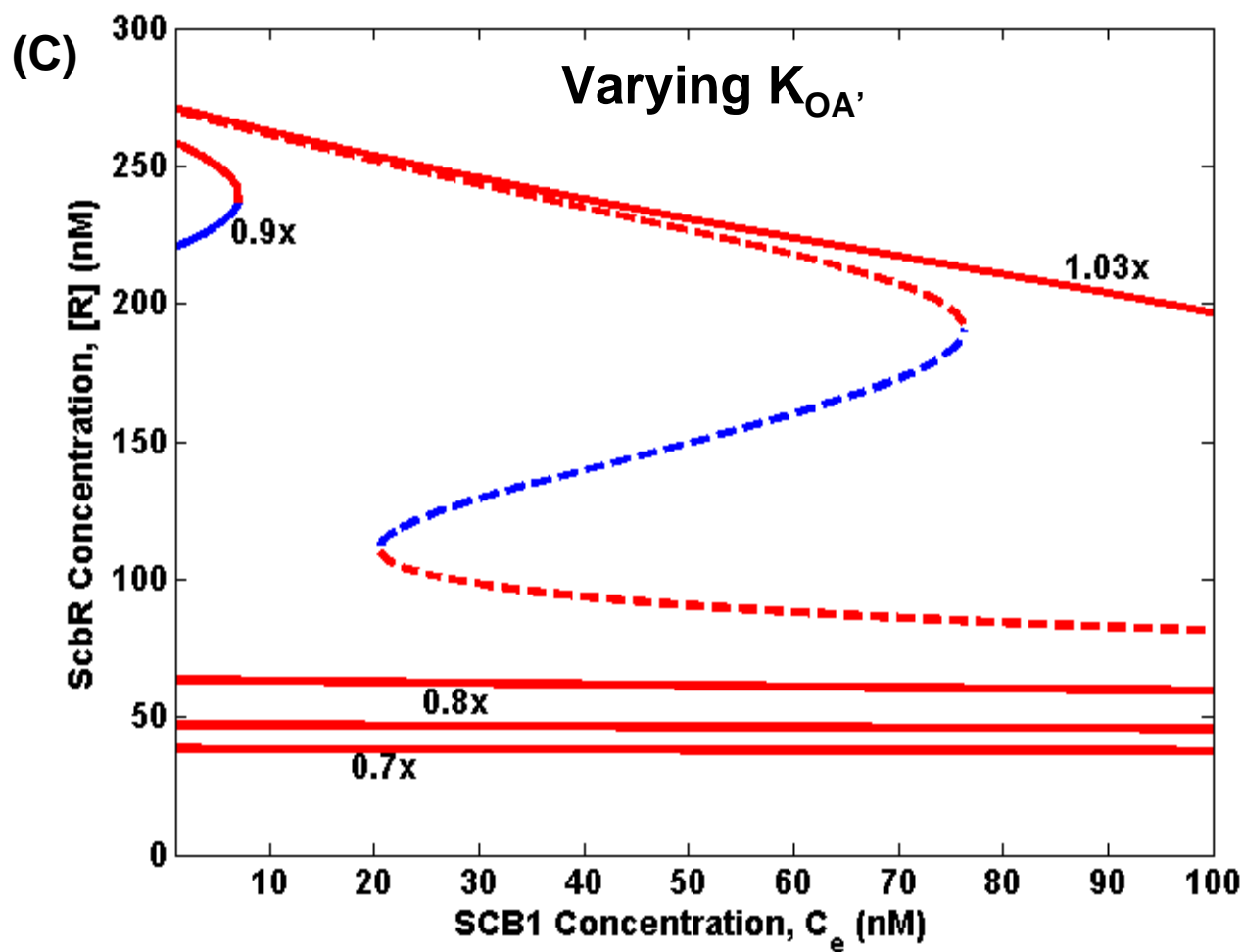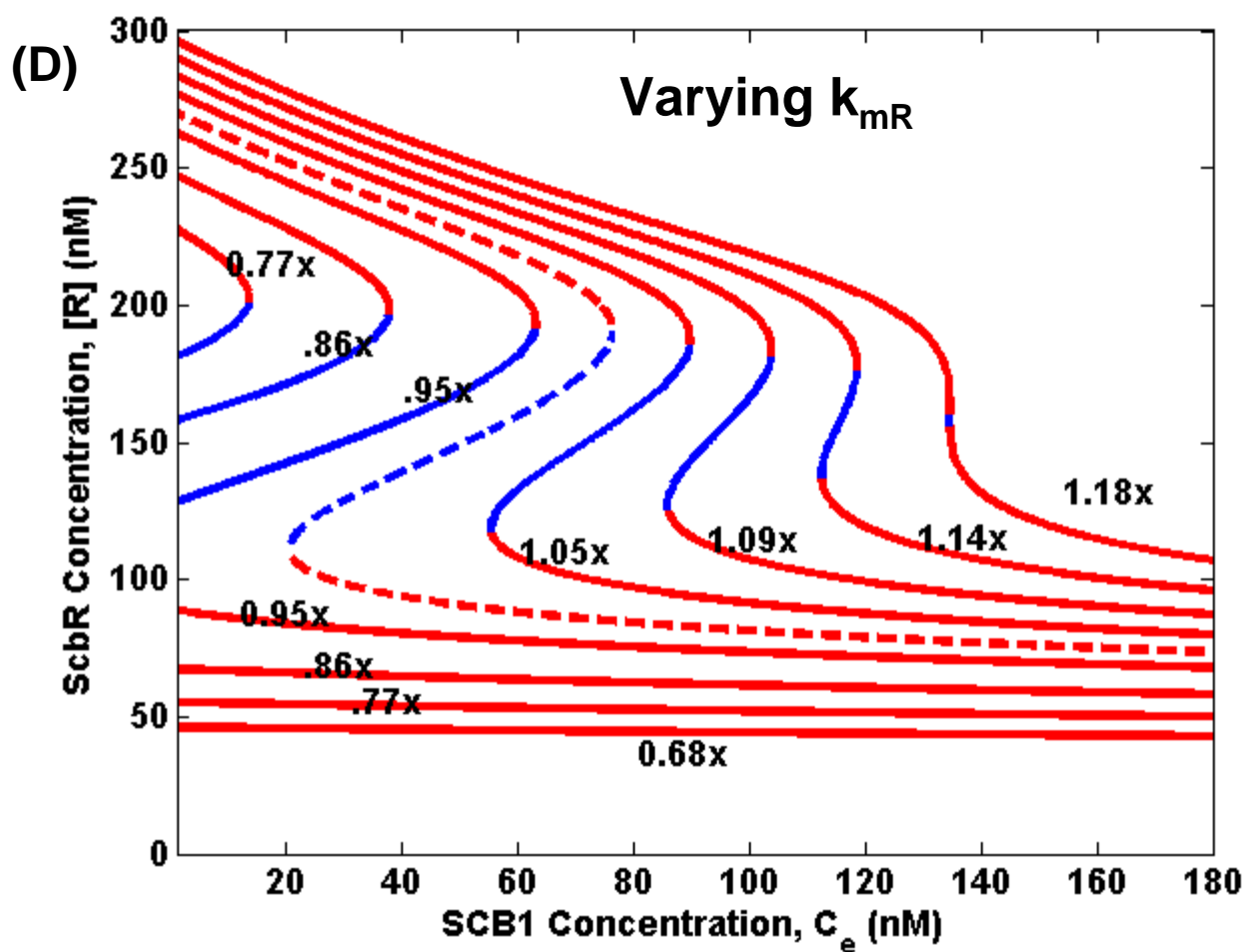

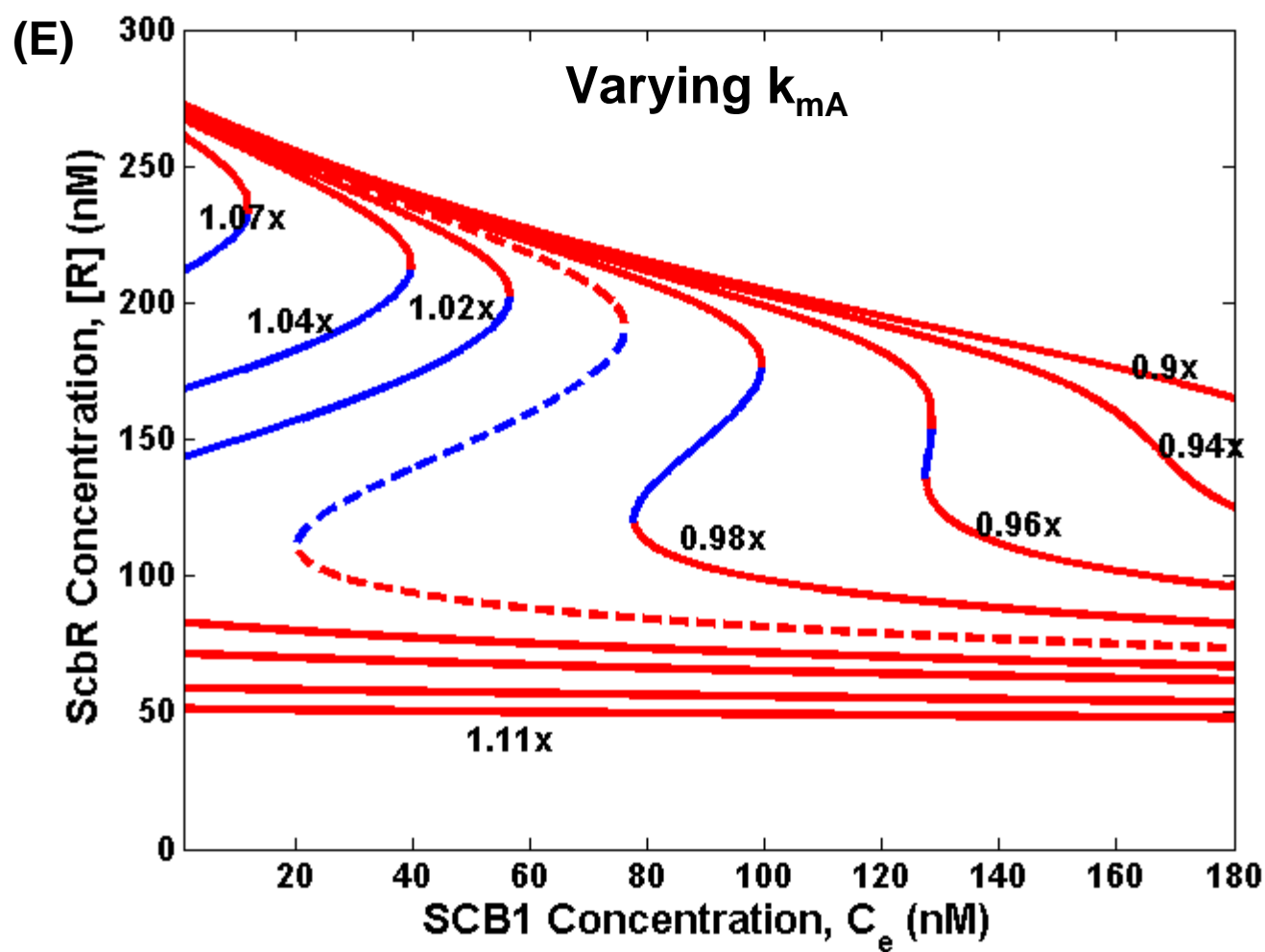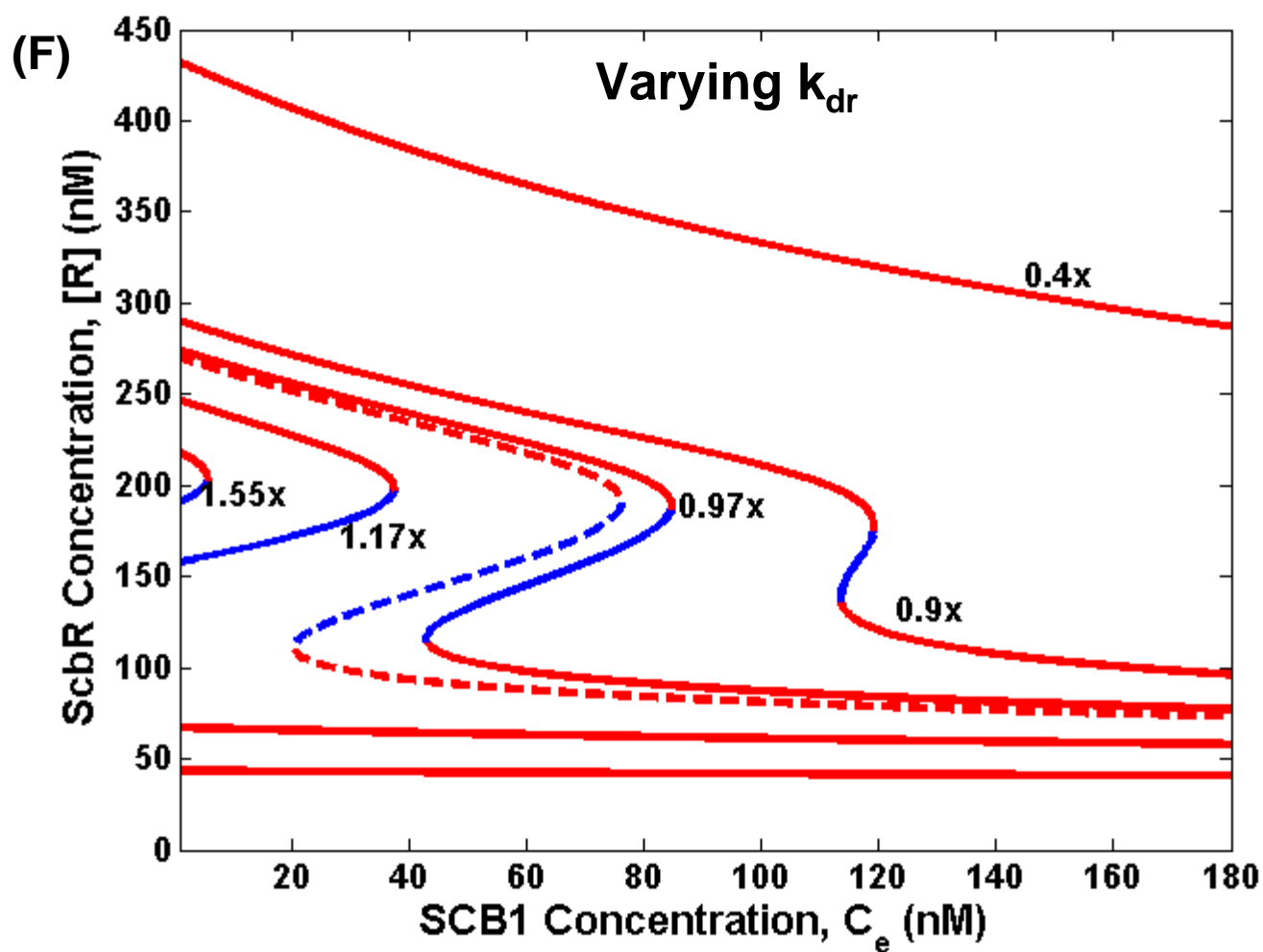

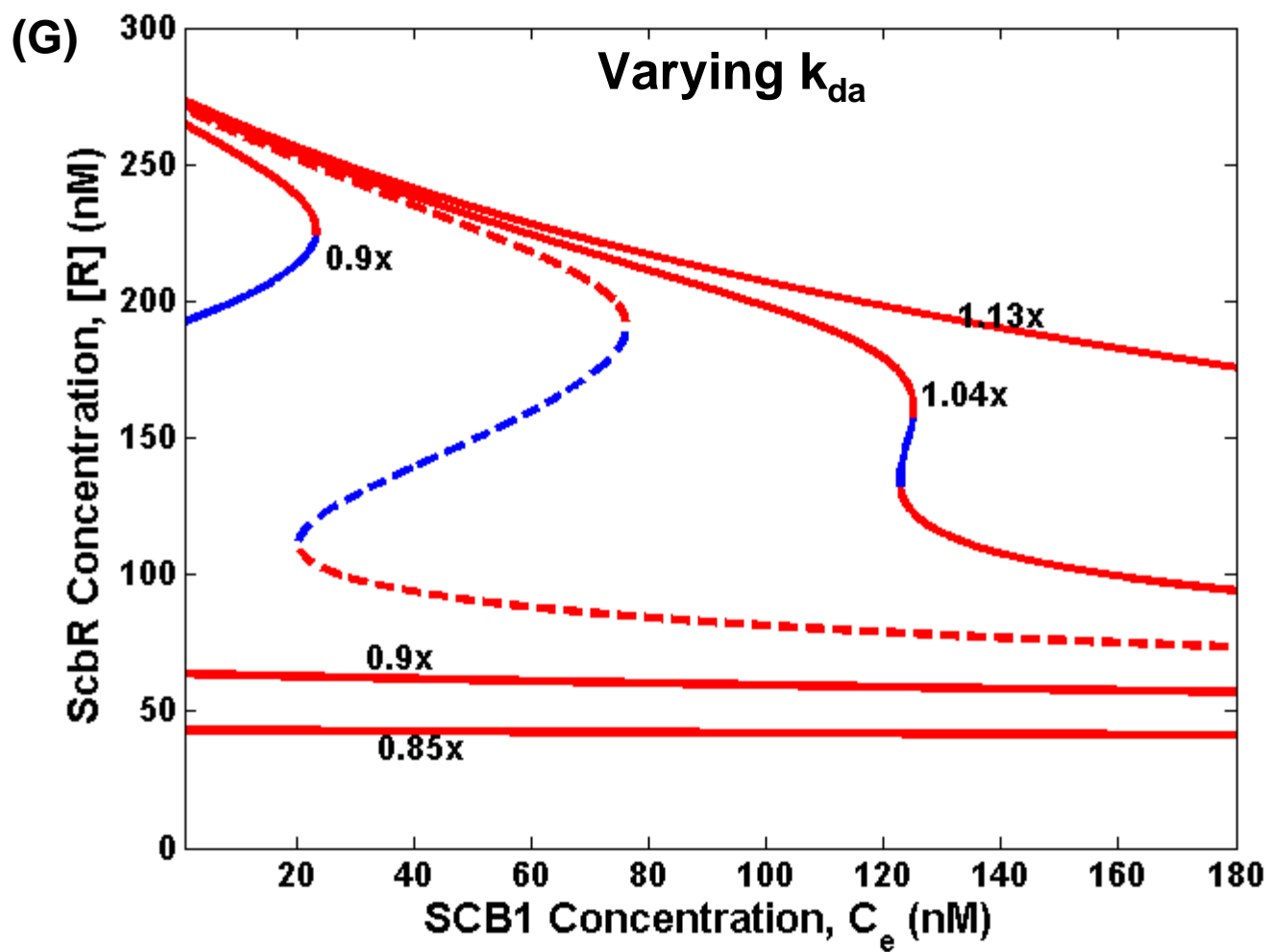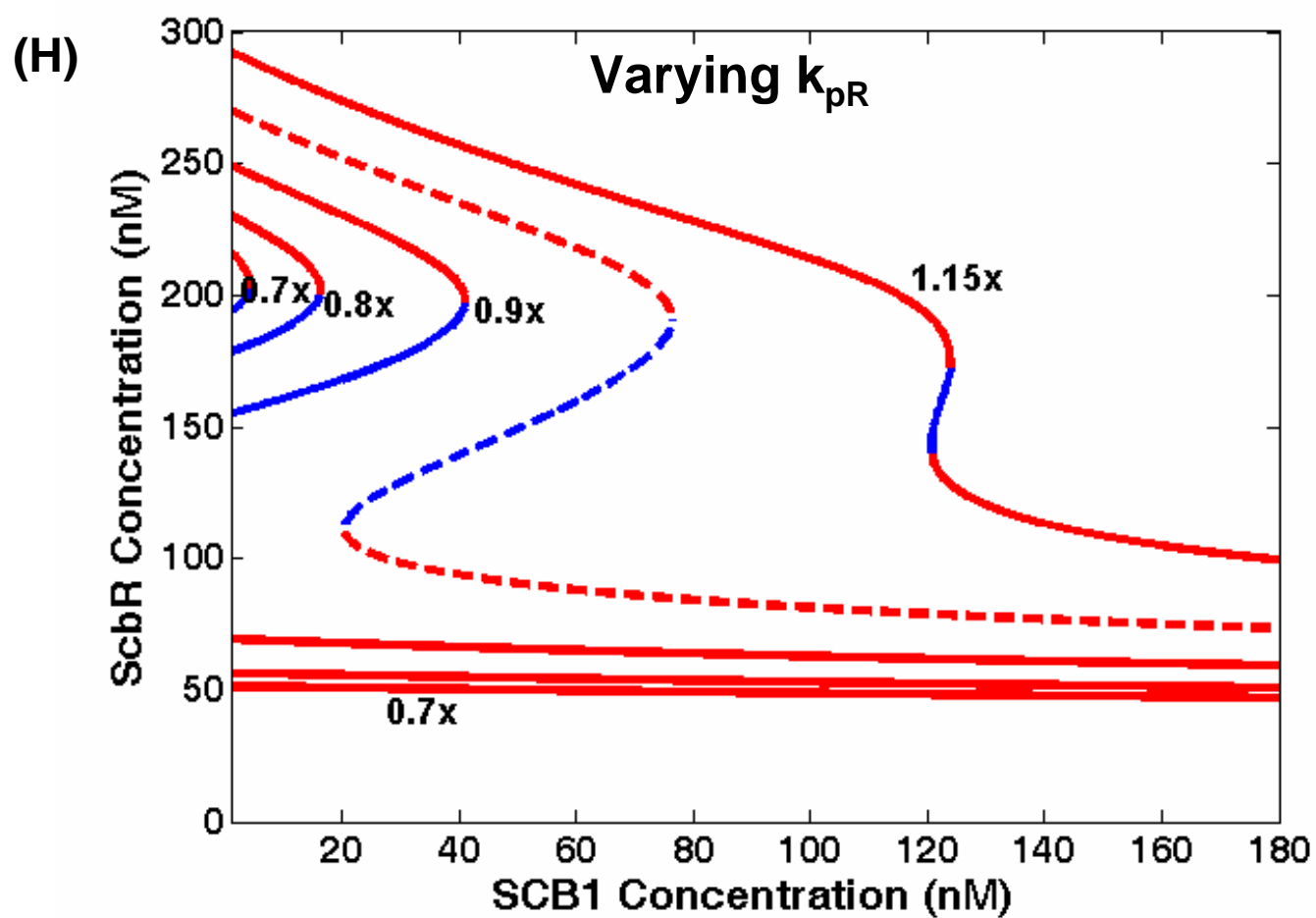

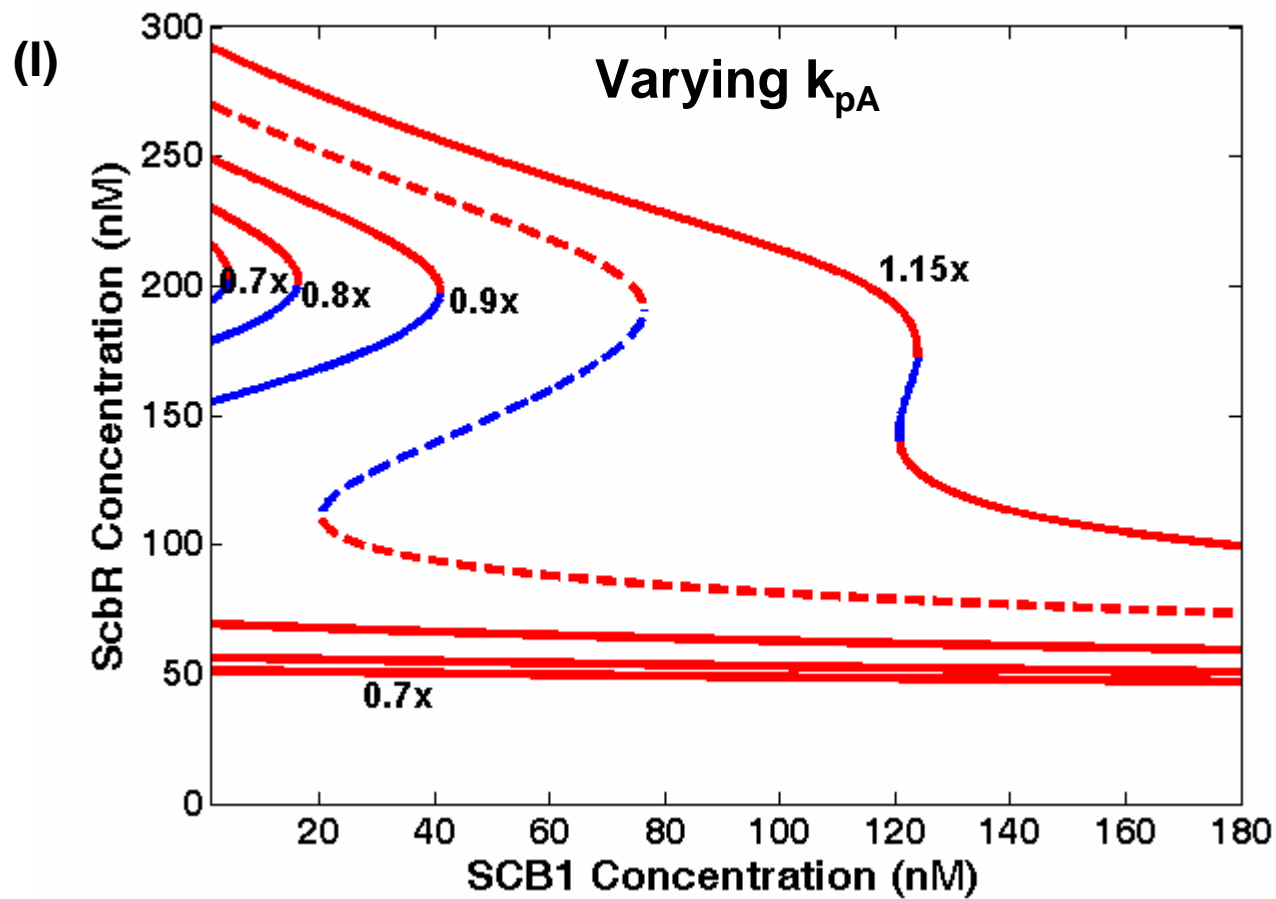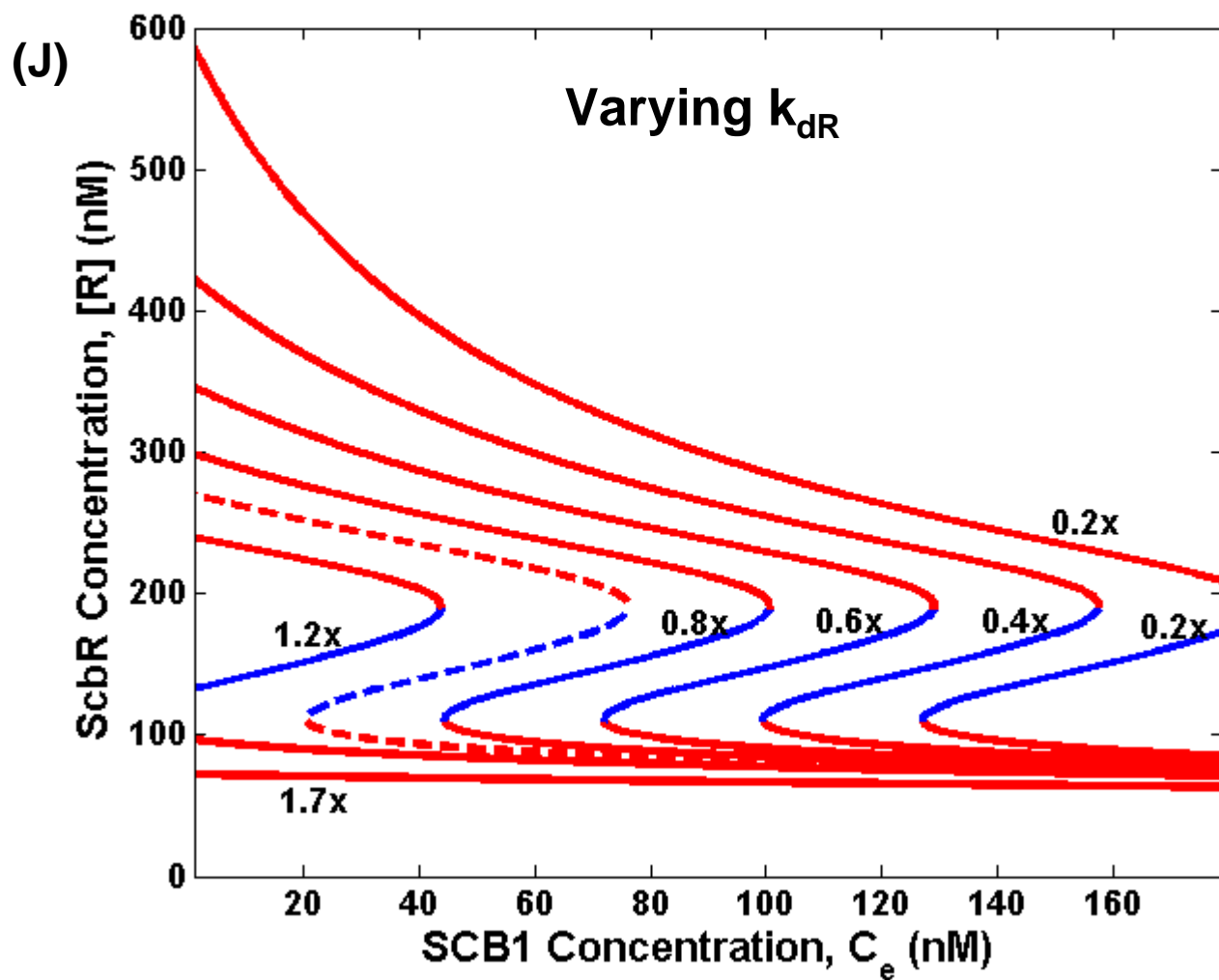

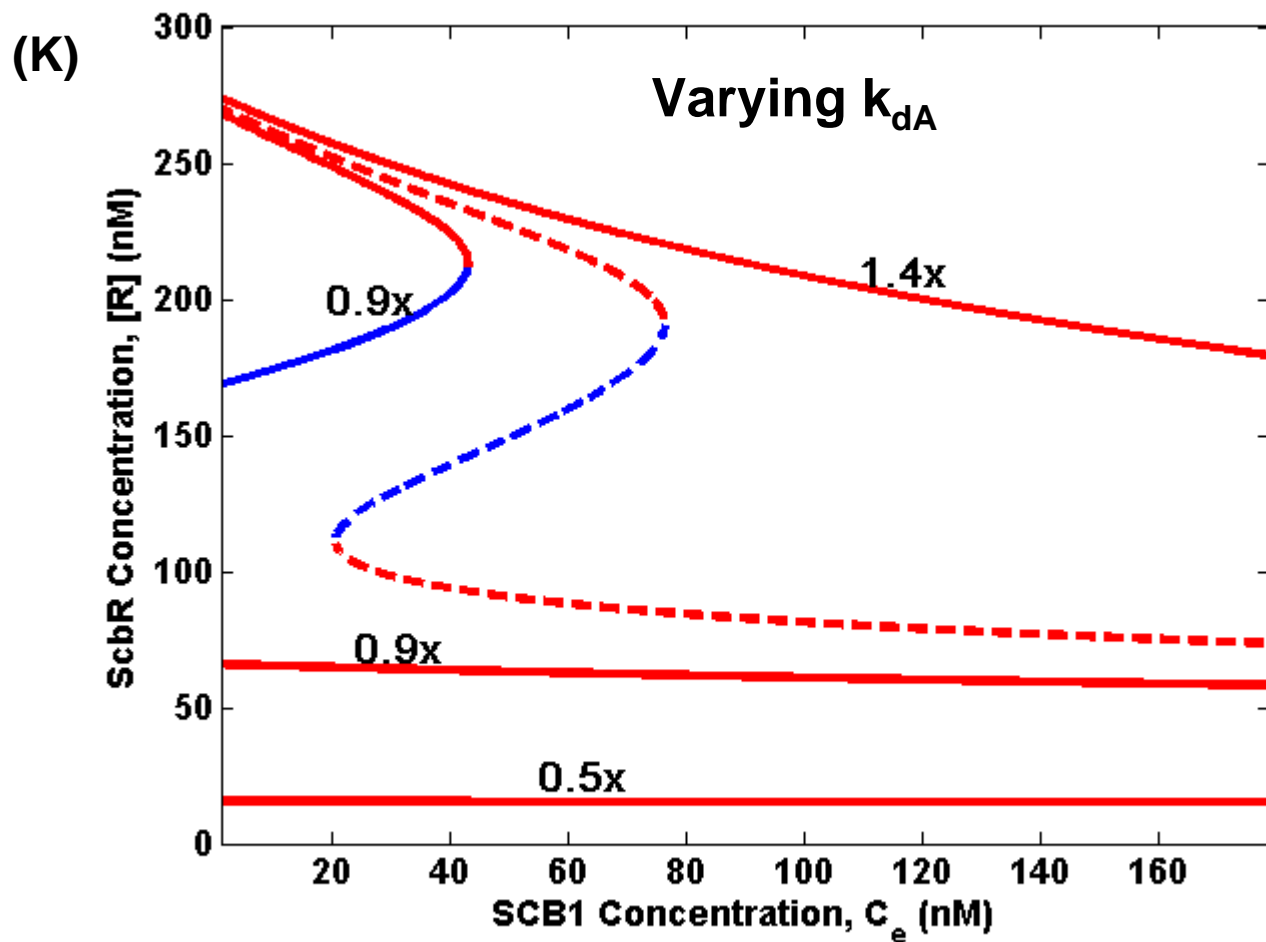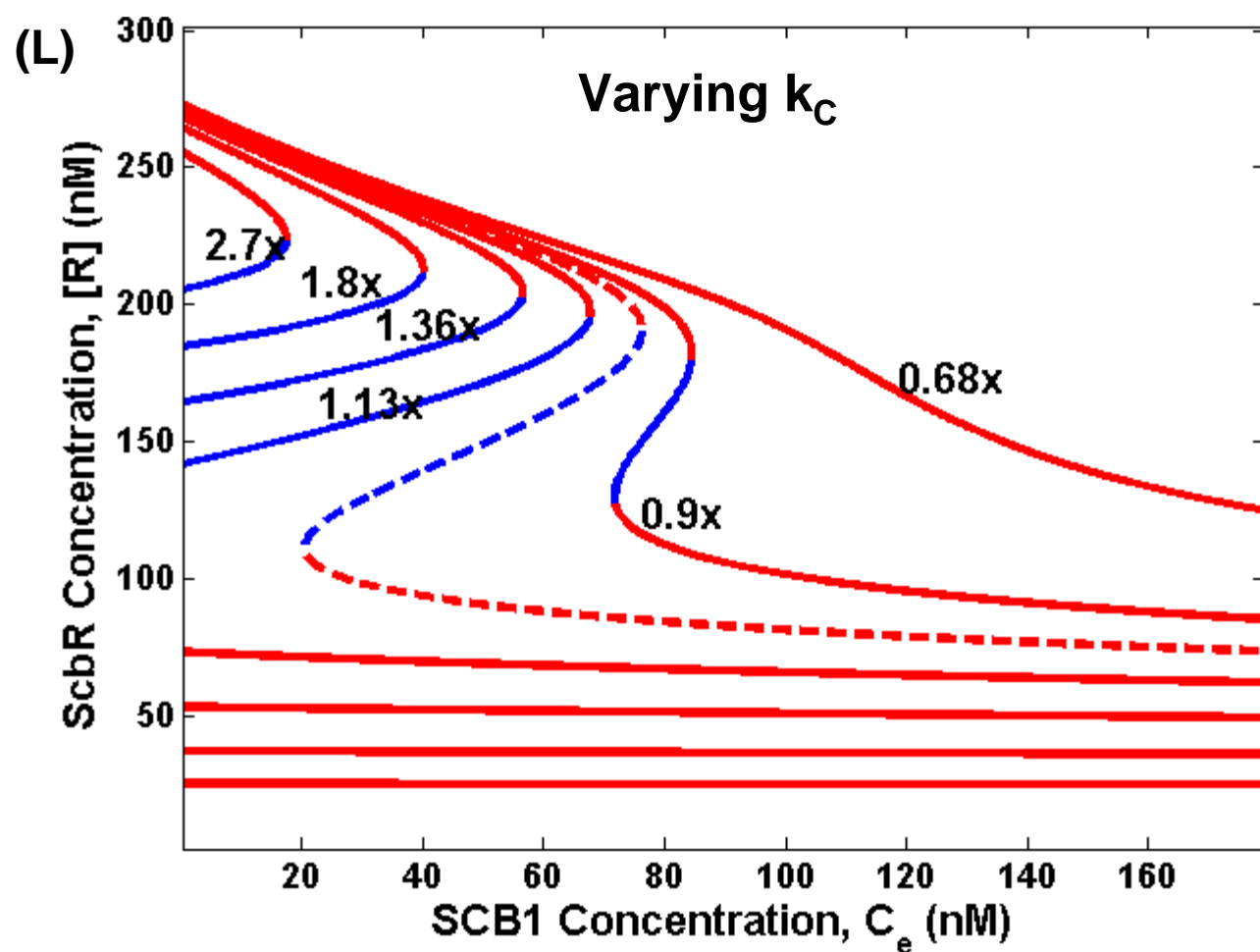

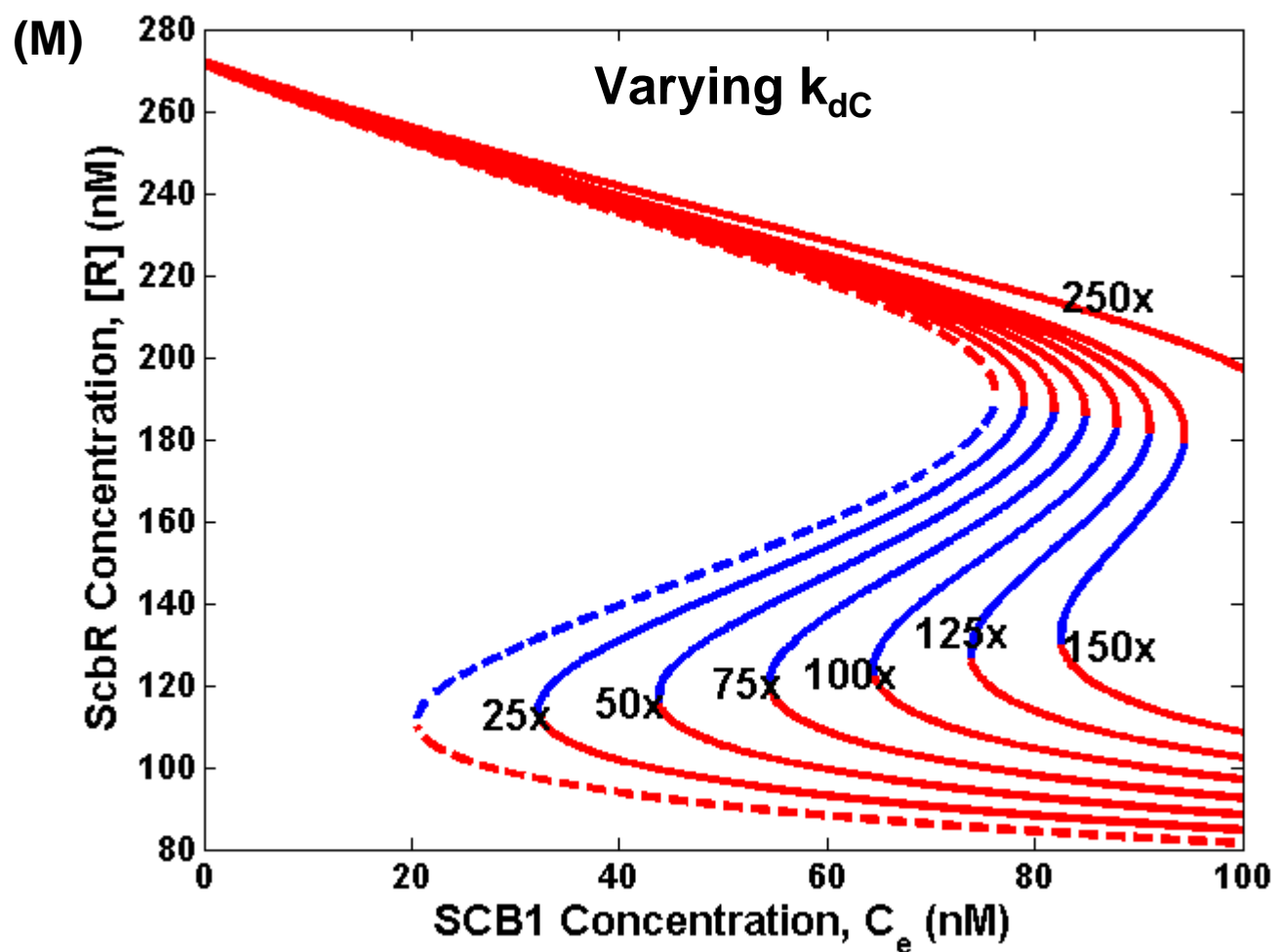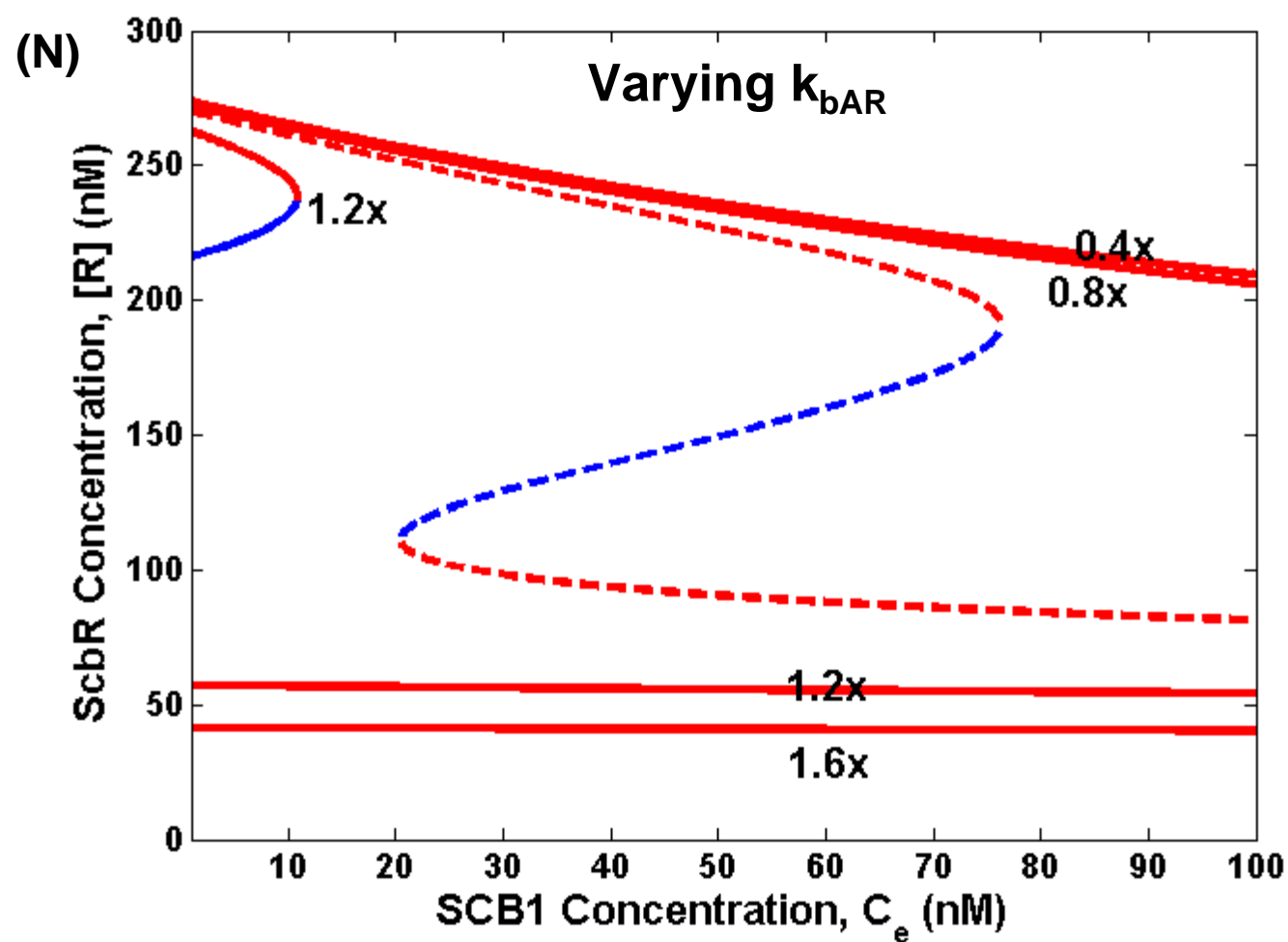

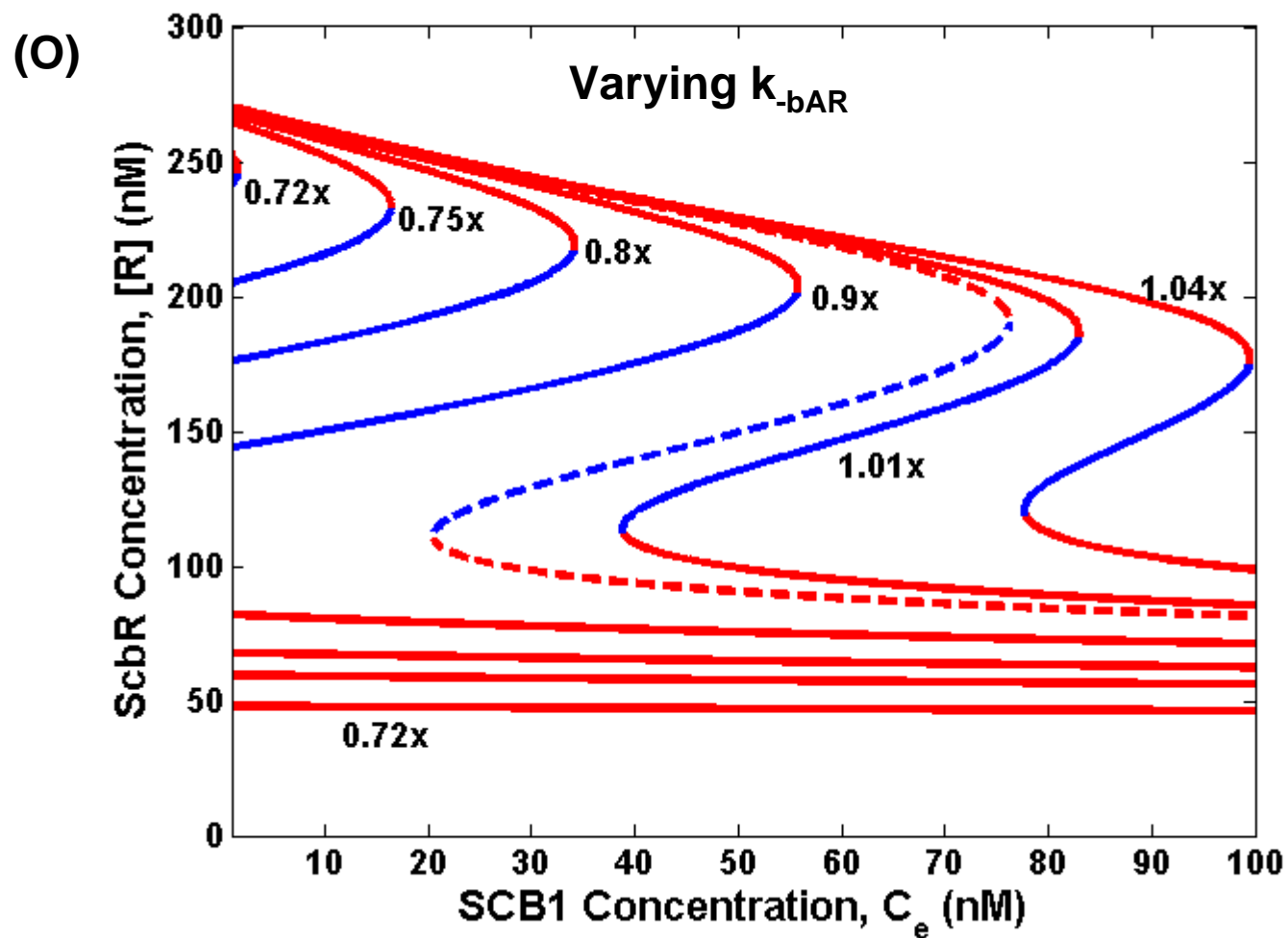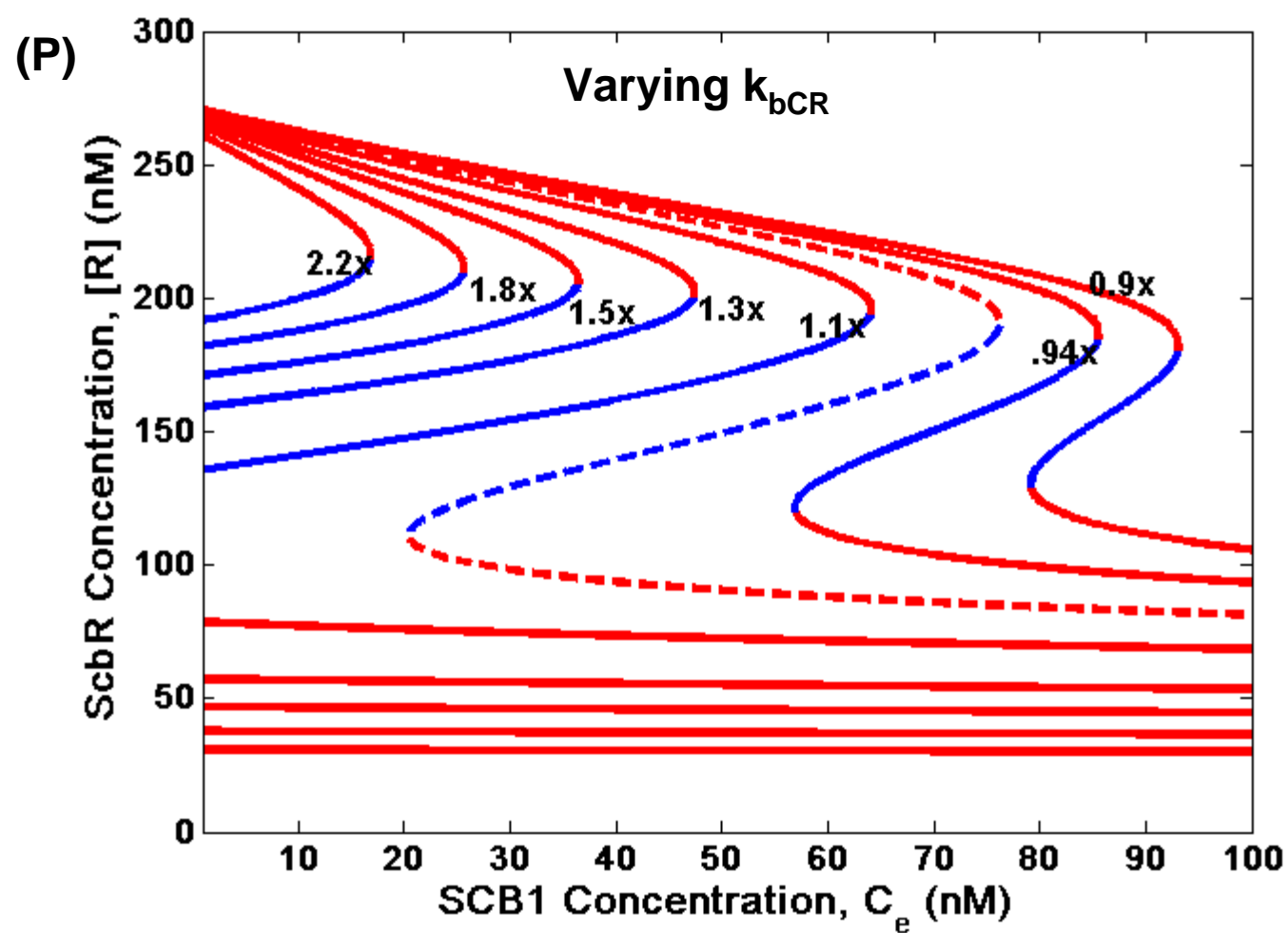

(Q)

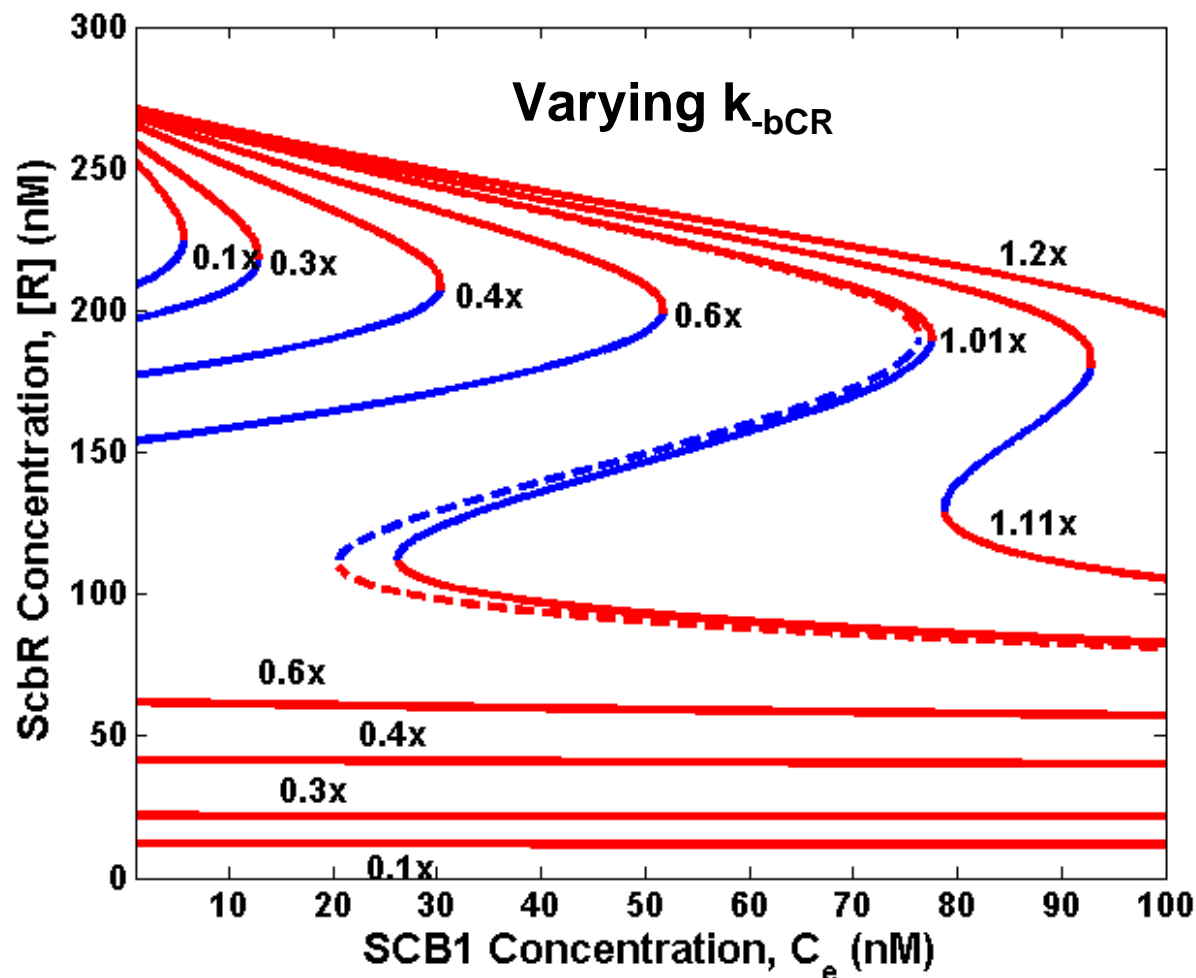

(R)

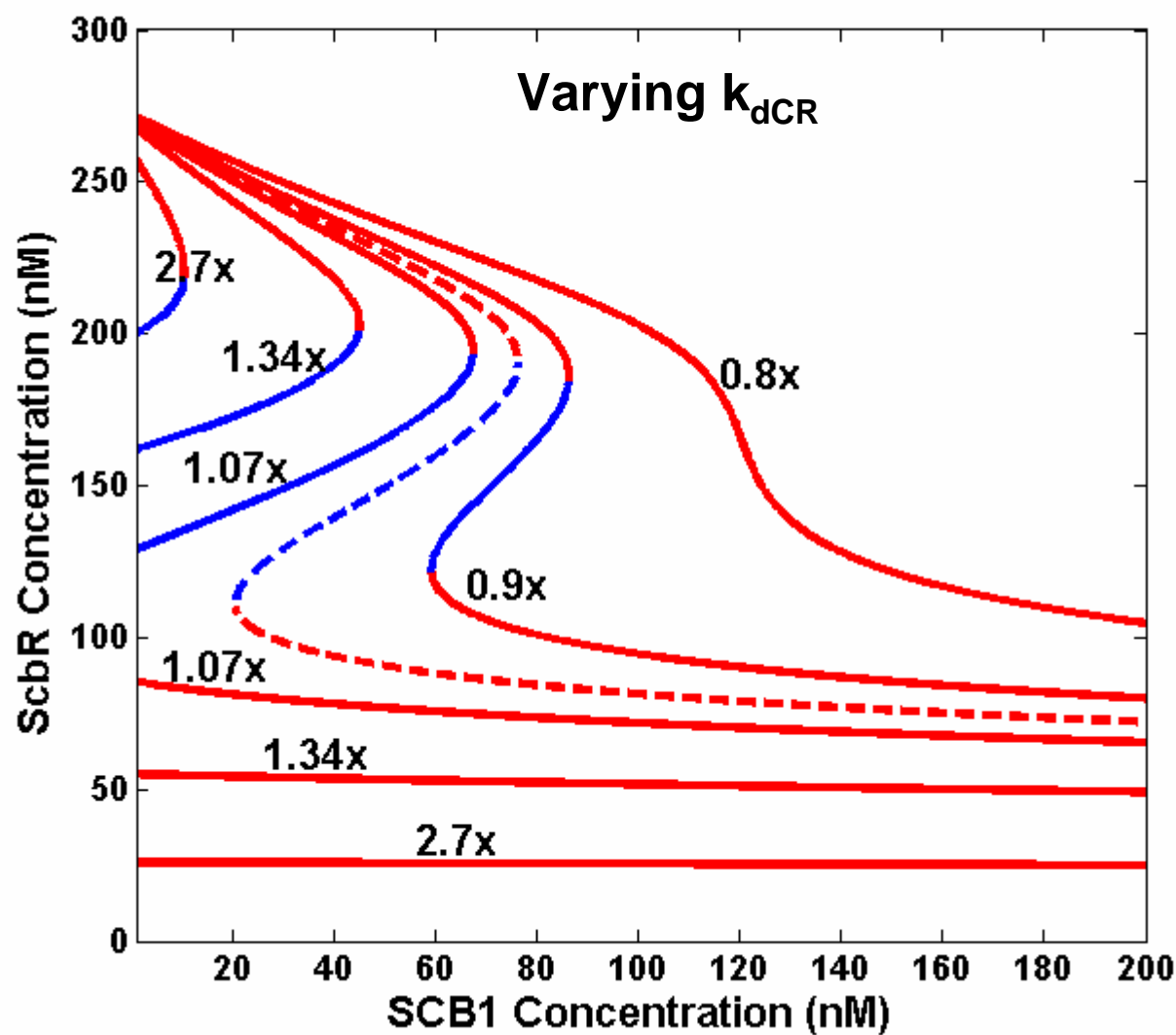

(S)

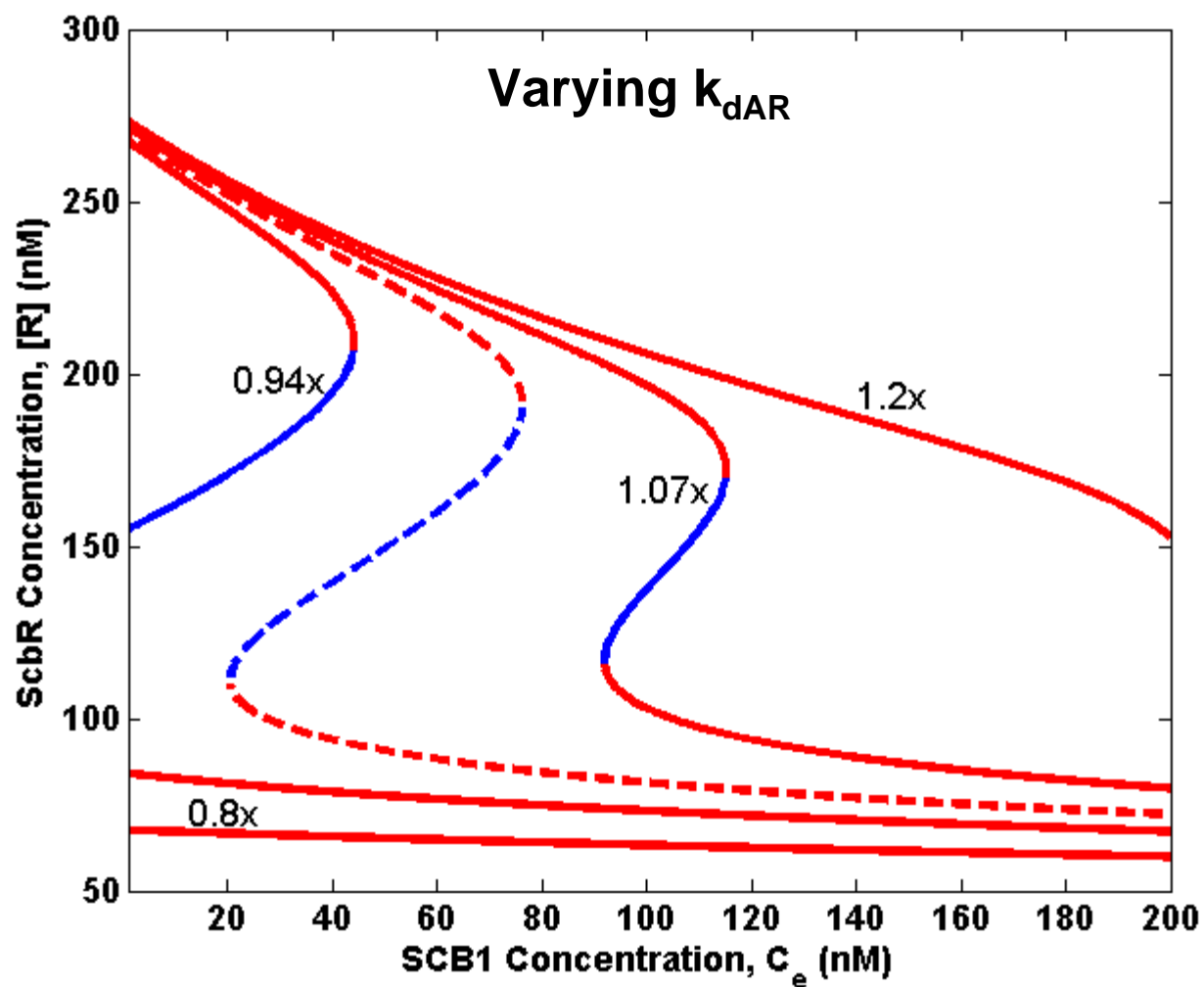

(T)

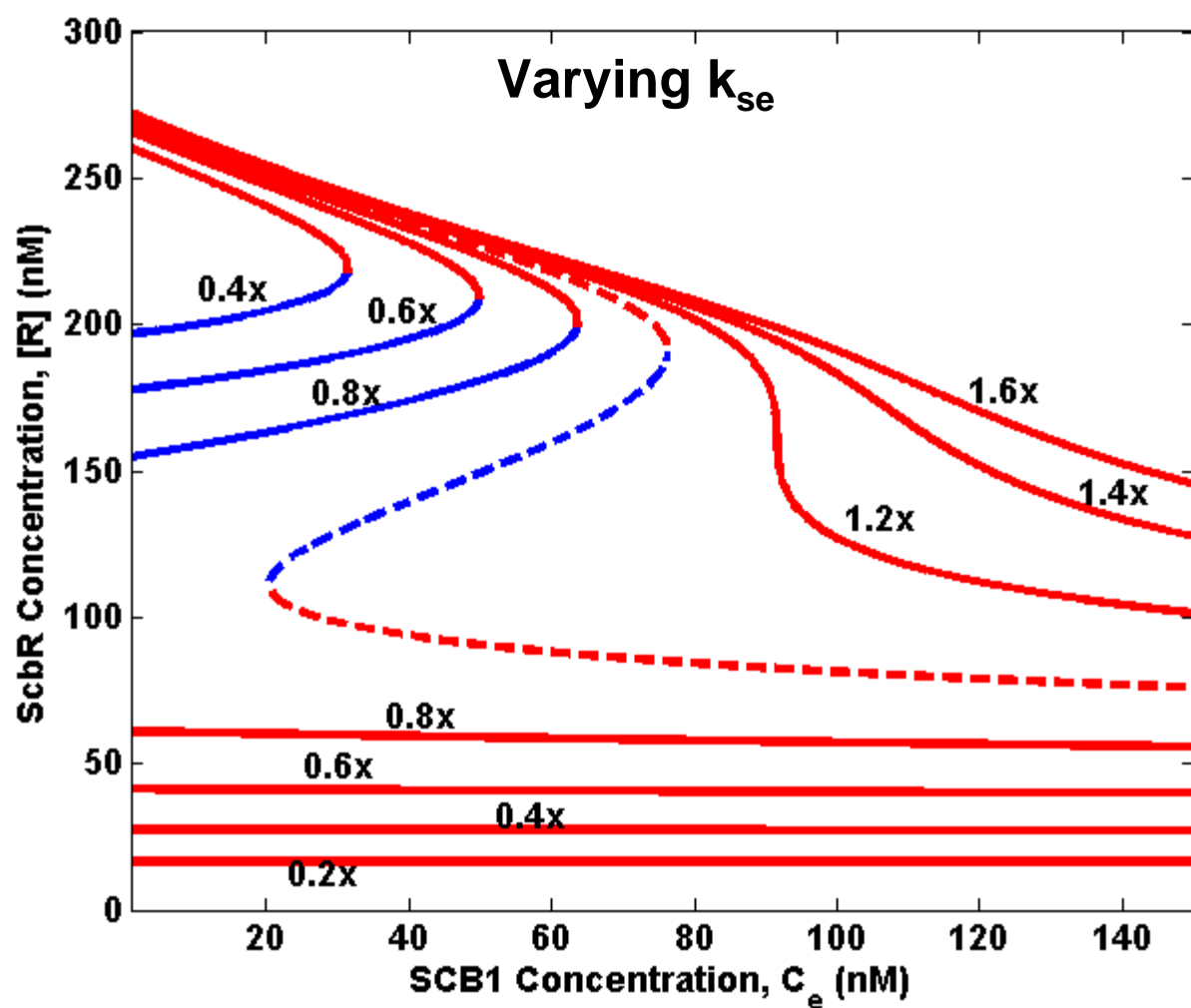

Supplement: Figure S2 — Effect of parameter perturbation on steady state response of butyrolactone system to constant extracellular SCB1. Each plot shows the results of simulation in which only one parameter is varied, keeping the rest constant at the nominal values listed in Table 2. The values of the parameter being varied are shown next to the respective steady state plot. The dashed line in each plot corresponds to the nominal parameter values. The parameter being varied is (A) KOR, (B) KOA, (C) KOA′, (D) kmR (E) kmA (F) kdr (G) kda (H) kpR (I) kpA (J) kdR (K) kdA (L) kC (M) kdC (N) kbAR (O) k−bAR (P) kbCR (Q) k−bCR (R) kdCR (S) kdAR (T) kse (0.15 MB PDF) [file pone.0002724.s002.pdf]

(A)

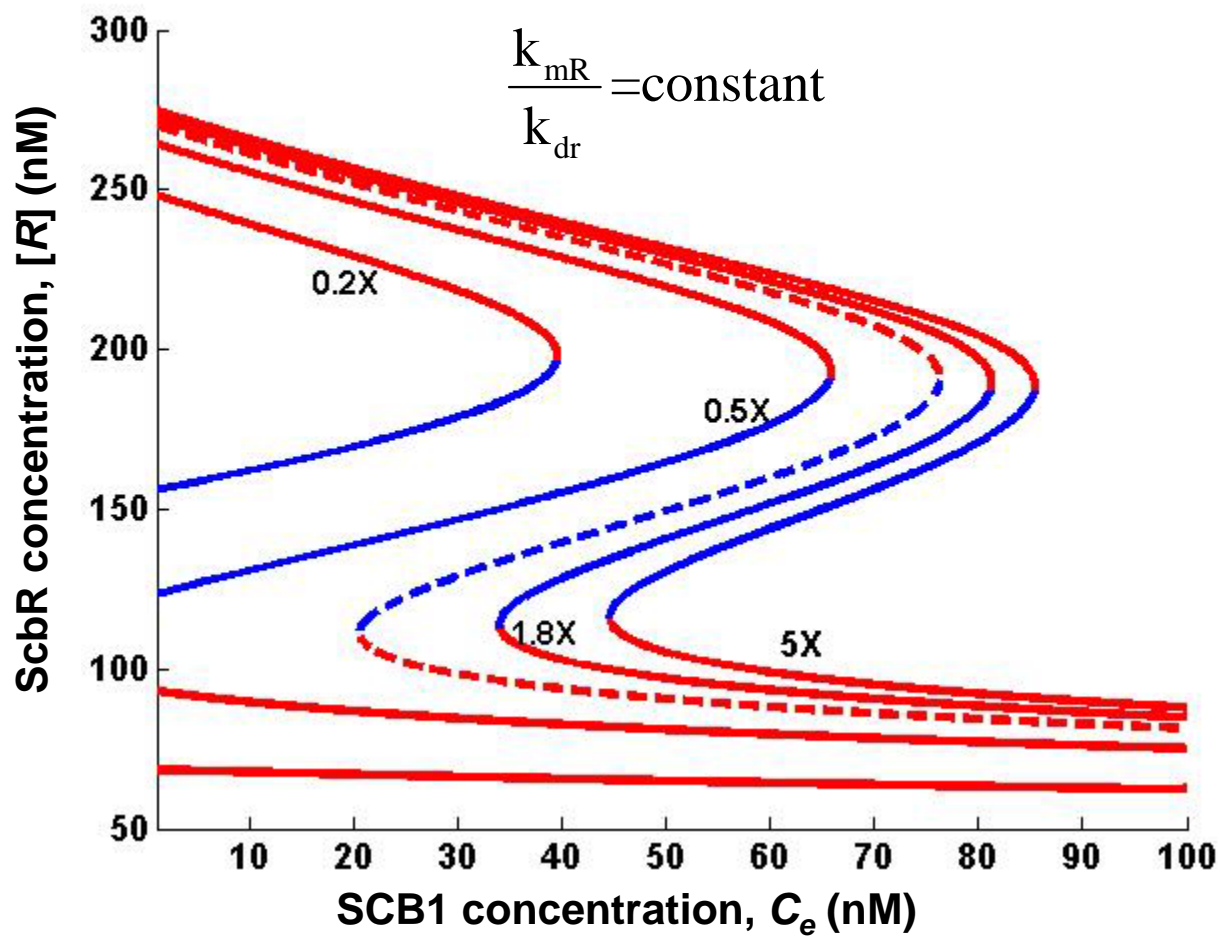

(B)

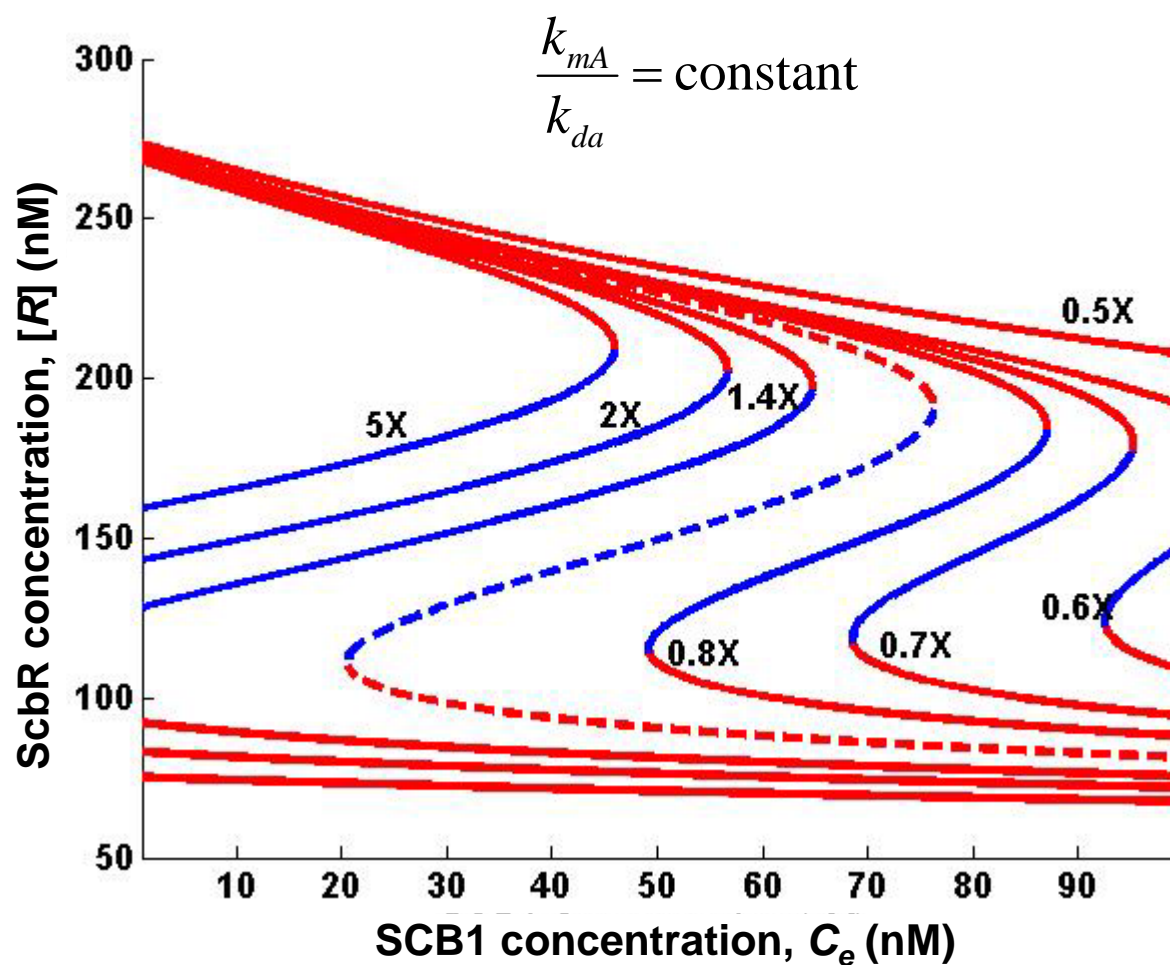

(C)

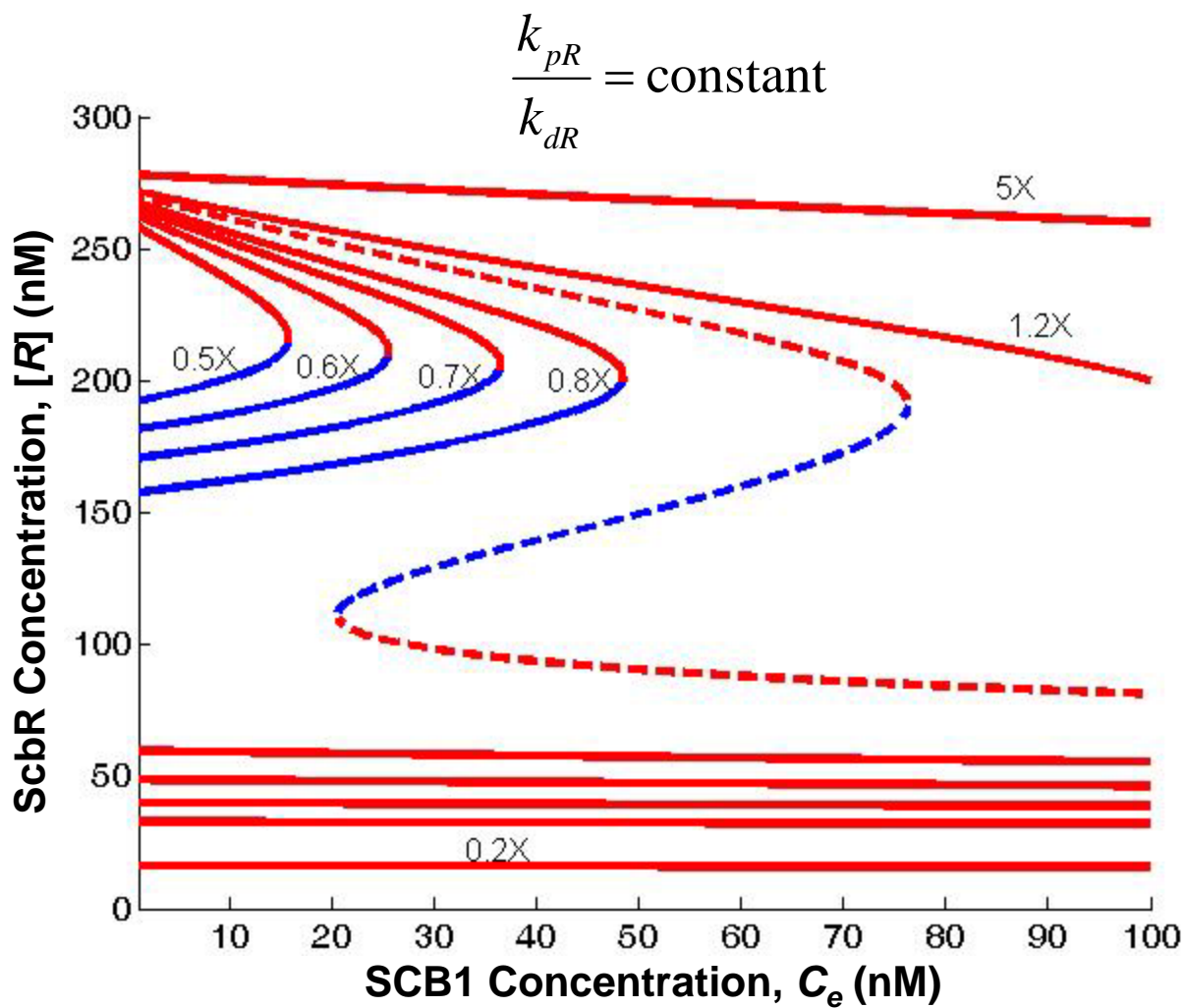

(D)

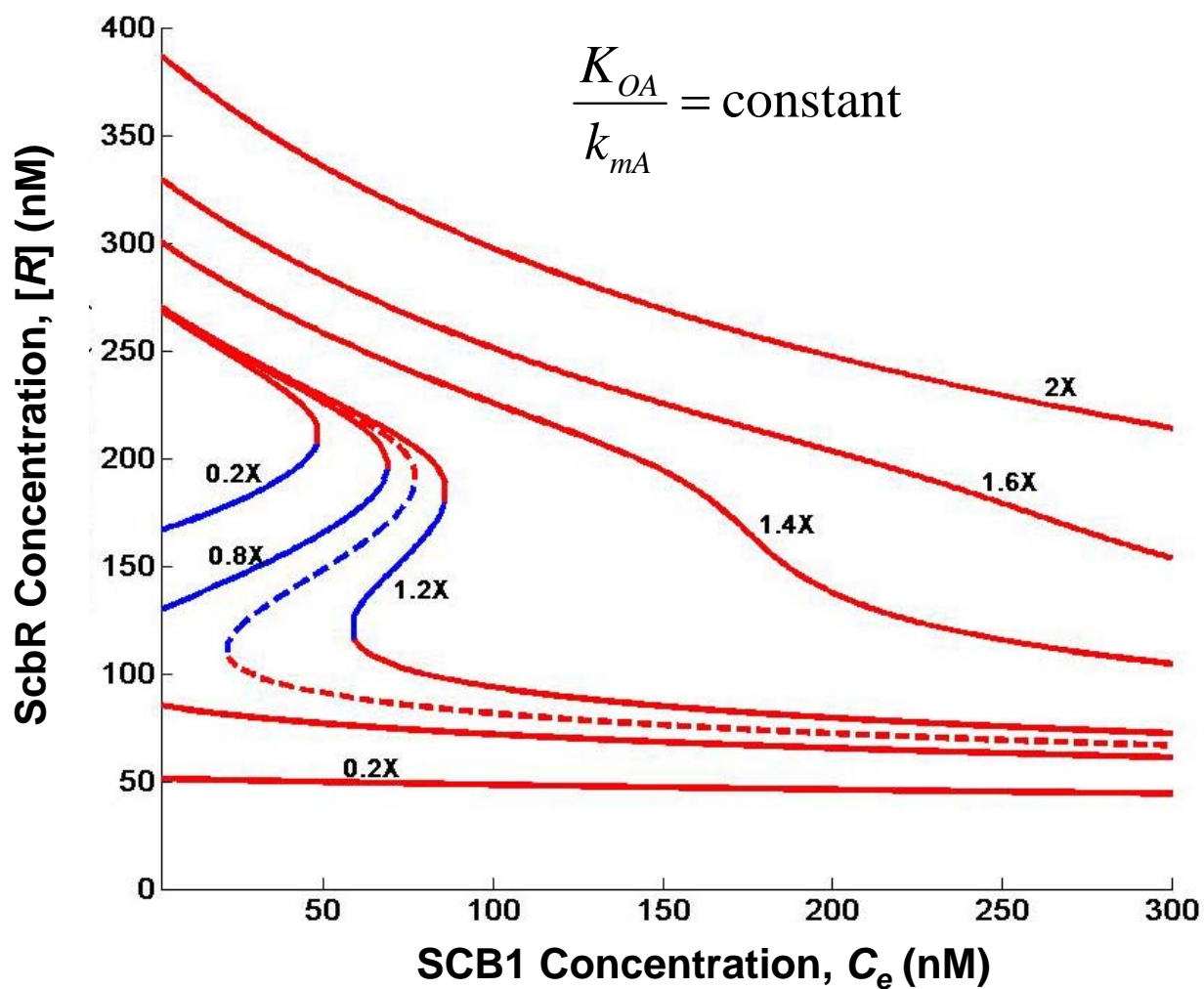

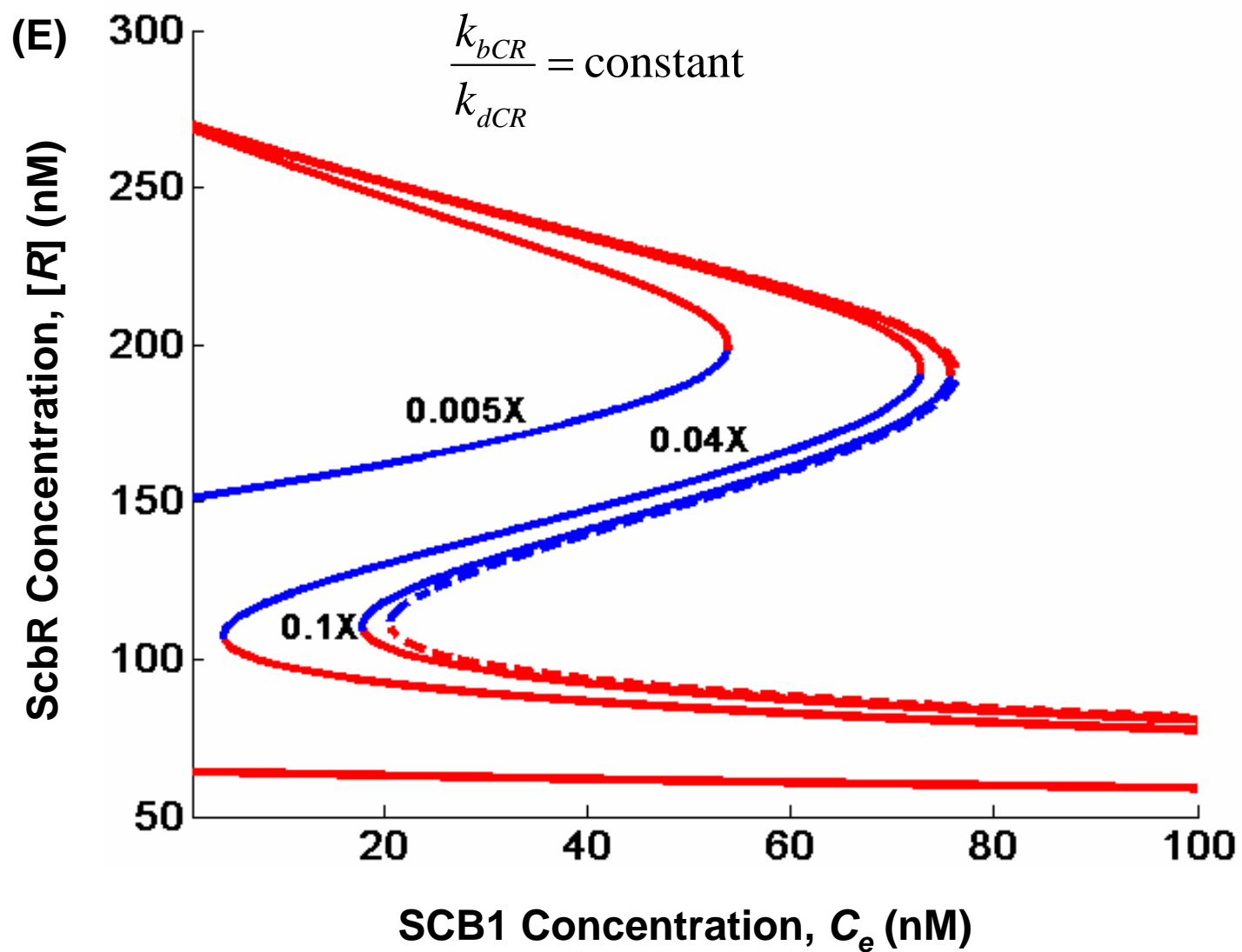

Supplement: Figure S3 — Effect of two parameter perturbation. For each simulation, two parameters are varied such that their ratio is constant. Dotted line corresponds to the nominal parameter values in Table 2. The two parameter combinations are (A) kmR/kdr, ratio of transcriptional rate constant to degradation rate constant of scbR mRNA. (B) kmA/kda, ratio of transcriptional rate constant to degradation rate constant of scbA mRNA, (C) kpR/kdR, ratio of translational rate constant to degradation rate constant of ScbR protein, (D) KOA/kmA, ratio of equilibrium binding constant of ScbR to OA operator to transcriptional rate constant of scbA mRNA and (E) kbCR/kdCR, ratio of rate constant for dissociation of CR complex to degradation rate constant of CR complex. (0.53 MB PDF) [file pone.0002724.s003.pdf]

(A)

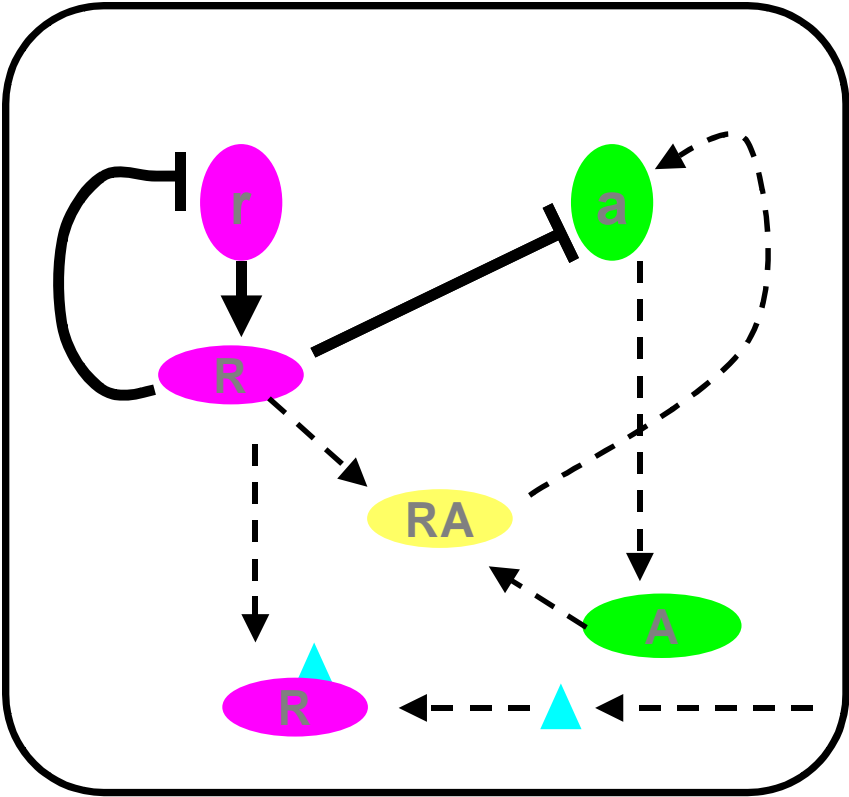

(B)

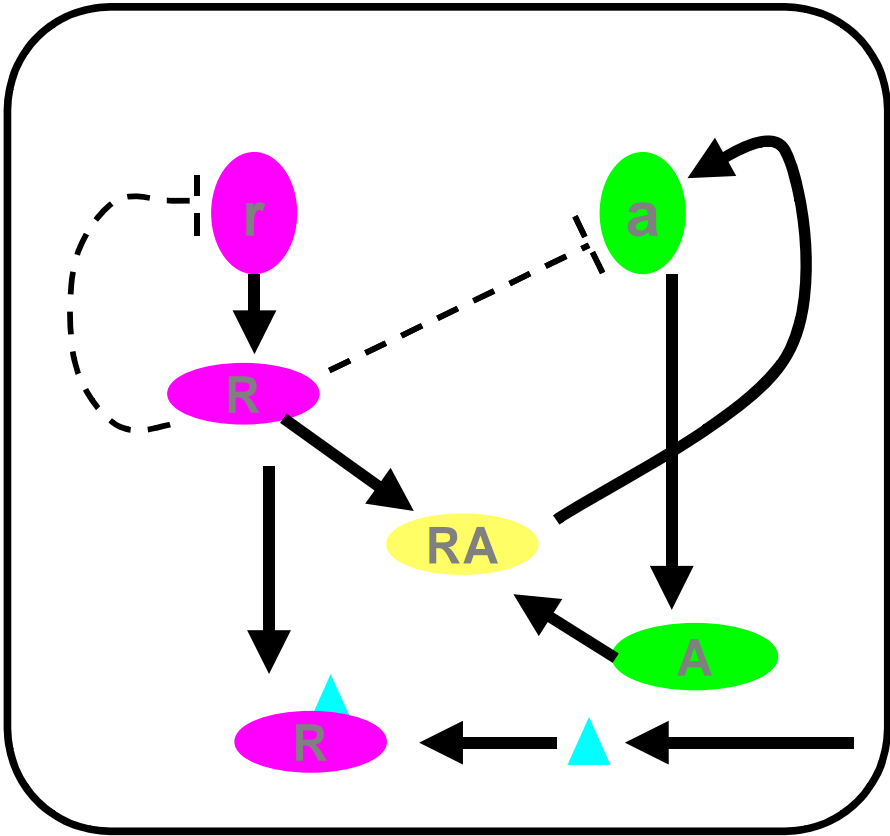

Supplement: Figure S4 — Physical description of the two steady states. (A) OFF state corresponding to high [R] or low [A]; (B) ON state corresponding to the low [R]. The solid line represents the dominant reactions in each steady state. Dashed lines show otherwise. (0.02 MB PDF) [file pone.0002724.s004.pdf]

(A)

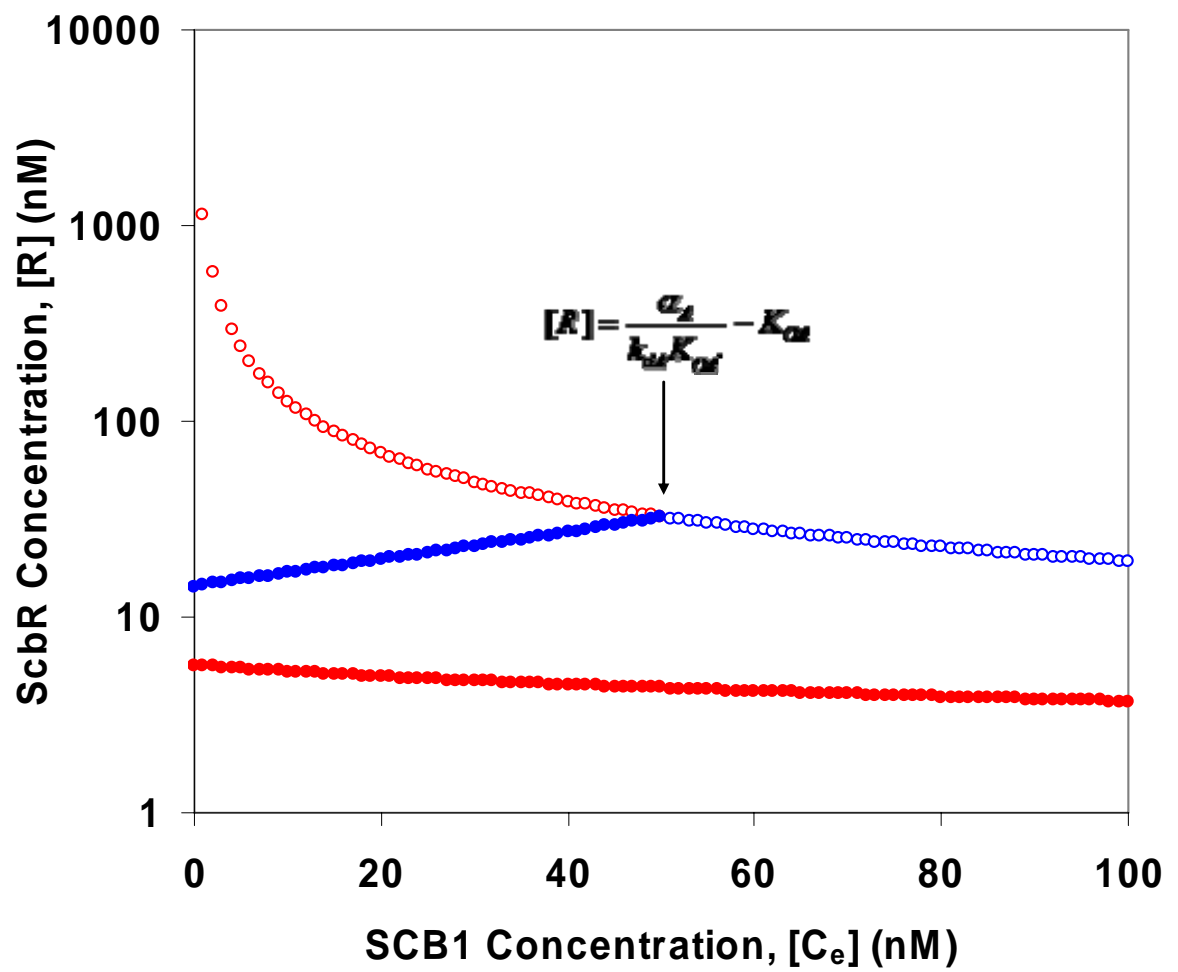

(B)

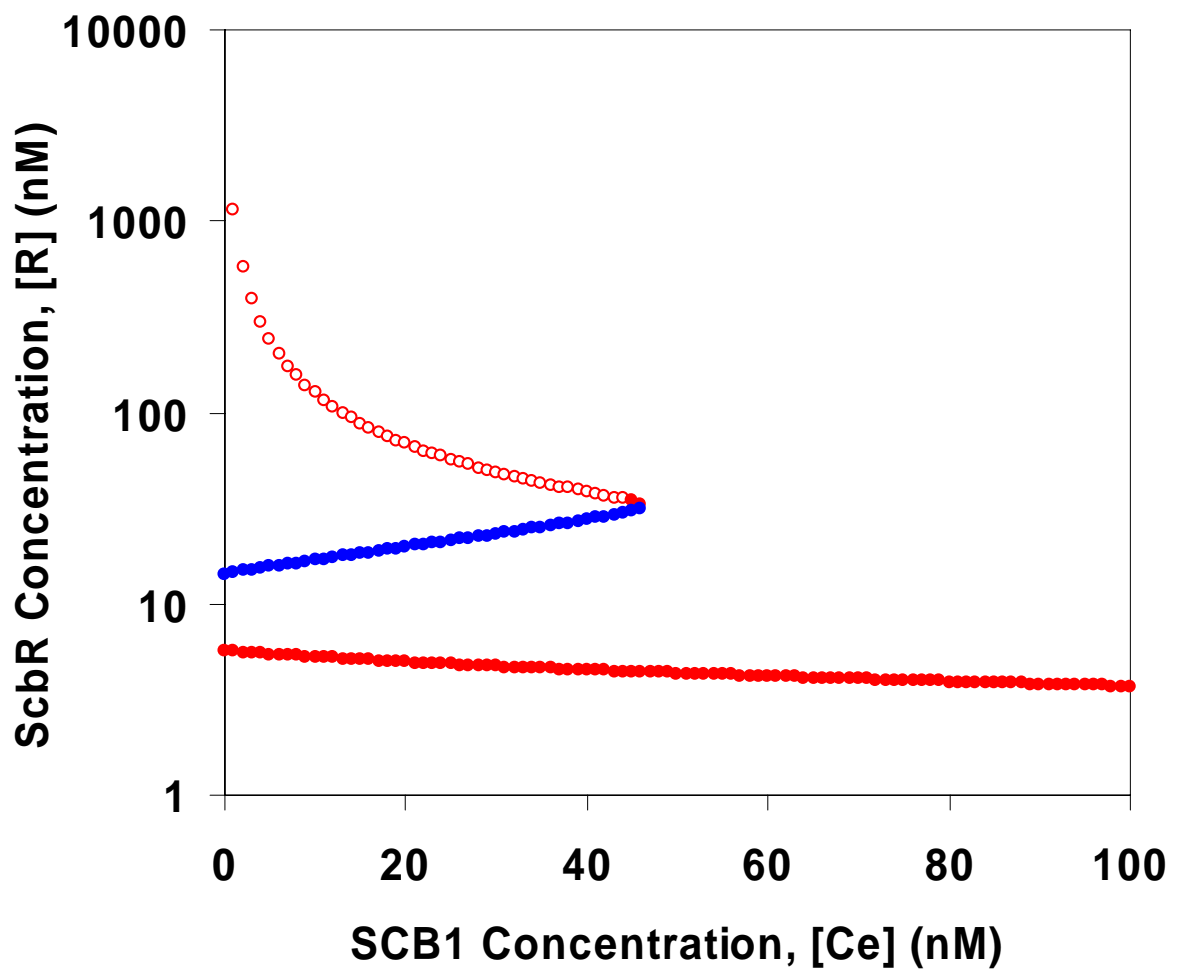

Supplement: Figure S5 — Steady state behavior of simplified ScbA/ScbR system. The steady state concentration of ScbR protein, [R], is plotted as a function of extracellular SCB1 concentration, Ce. (A) Steady state plot when basal transcription rate of scbA is neglected. (B) Steady state plot when scbA is transcribed at a basal rate. Stable steady states are denoted in red, while unstable steady states are marked in blue. Open circles denote the steady state corresponding to [A] = 0, whereas filled circles denote the steady states corresponding to [A]≠0. The transition point beyond which the fixed state corresponding to [A] = 0 becomes unstable is marked by an arrow. At this point, one of the fixed roots corresponding to also disappears. (0.04 MB PDF) [file pone.0002724.s005.pdf]
