# Supplementary material for: A Bistable Gene Switch for Antibiotic Biosynthesis: The Butyrolactone Regulon in Streptomyces coelicolor
Source: PLoS One. 2008 Jul 16;3(7):e2724. doi: 10.1371/journal.pone.0002724 (PMC2444045; doi:10.1371/journal.pone.0002724)
Supplement: Text S1 — Analytical steady state analysis for a simplified ScbA/ScbR system (0.14 MB DOC) [file pone.0002724.s006.doc]

To look for conditions for bistability, a simplified system is studied where ScbA protein can bind to its operator $O_{A^{'}}$ and activate its own transcription. This obviates the requirement of formation of ScbA-ScbR complex that activates *scbA*. In addition, the growth rate is embedded in the decay rates of all species, and the basal rate of production of *scbA* is neglected. The degradation of ScbR protein is also neglected. The resulting set of equations is listed below:

$\frac{d[r]}{dt}=k_{mR}\frac{K_{OR}}{K_{OR}+[R]}-k_{dr}[r]$ (1)

$$\frac{d[a]}{dt}=k_{mA}\frac{K_{OA}*[A]}{(K_{OA}+[R])(K_{OA^{'}}+[A])}-k_{da}.[a]$$

$$\frac{d[R]}{dt}=k_{pR}[r]-k_{bCR}[C_{i}][R]+k_{-bCR}[CR]$$

$$\frac{d[A]}{dt}=k_{pA}.[a]-k_{dA}.[A]$$

$$\frac{d[CR]}{dt}=k_{bCR}[C_{i}][R]-(k_{-bCR}+k_{dCR})[CR]$$

$$\frac{d[C_{i}]}{dt}=k_{C}.[A]-(k_{bCR}.[C_{i}].[R]-k_{-bCR}.[CR])-k_{se}.([C_{i}]-[C_{e}])$$

To determine the steady state solutions, the L.H.S of the above equations is set to zero.

$$[r]=\frac{k_{mR}}{k_{dr}}\frac{K_{OR}}{K_{OR}+[R]}$$

$$[a]=\frac{k_{mA}}{k_{da}}\frac{K_{OA}*[A]}{(K_{OA}+[R])(K_{OA^{'}}+[A])}$$

$[CR]=\alpha_{CR}[C_{i}][R]$ where $\alpha_{CR}=\frac{k_{dCR}k_{bCR}}{(k_{-bCR}+k_{dCR})}$

$\frac{\alpha_{R}}{K_{OR}+[R]}=\alpha_{CR}[C_{i}][R]$, where $\alpha_{R}=\frac{k_{pR}k_{mR}KOR}{k_{dr}}$

$\frac{\alpha_{A}*[A]}{(K_{OA}+[R])(K_{OA^{'}}+[A])}=k_{dA}.[A]$ where $\alpha_{A}=\frac{k_{pA}.k_{mA}.K_{OA}}{k_{da}}$

$k_{C}.[A]=\alpha_{CR}[C_{i}][R]+k_{se}.([C_{i}]-[C_{e}])$.

Equation (1.11) suggests that one steady state of the system is when $[A]=0$. The corresponding value for $[C_{i}]\text{ is }\frac{k_{se}[C_{e}]}{\alpha_{CR}[R]+k_{se}}$. [*R*] is given by the roots of the following cubic equation:

$$\begin{matrix} k_{dR}\alpha_{CR}[R]^{3}+(k_{dR}K_{OR}\alpha_{CR}+k_{dR}k_{se}+\alpha_{CR}k_{se}[C_{e}])[R]^{2}+(\alpha_{CR}k_{se}[C_{e}]K_{OR}+k_{dR}K_{OR}k_{se}-\alpha_{CR}k_{pR}K_{OR})[R] \\ -k_{se}k_{pR}K_{OR}=0 \end{matrix}$$

Physiologically, the steady state with [*A*] = 0 corresponds to the OFF state where a high concentration of ScbR protein represses transcription of *scbA*. This results in low levels of *CR* complex. The dominant characteristic of the system in this state is that the auto-repressed synthesis of ScbR is balanced by the low levels of CR complex formation. The stability condition for this steady state is:

$$k_{da}k_{dA}-\frac{k_{mA}k_{pA}}{K_{OA'}}\left(\frac{K_{OA}}{K_{OA}+[R]}\right)>0$$

For a fixed set of parameter values, the coefficients of equation 1.13 are a function of [*C_e_*]. [*R*] is observed to decrease with increasing *C_e_*. At low *C_e_*, [*R*] is high and the above stability condition is satisfied. However, this steady state is unstable if$[R]<\frac{\alpha_{A}}{k_{dA}K_{OA'}} -K_{OA}$.

The other steady states corresponding to $[A]\neq0$can be determined by solving the following equations based on 1.10-1.12 above:

$$\frac{\alpha_{A}}{k_{dA}(KOA+[R])}=(KOA^{'}+[A])$$

$$k_{C}.[A]=\alpha_{CR}[C_{i}][R]+k_{se}.[C_{i}]-k_{se}.[C_{e}]$$

$$\alpha_{R}=\alpha_{CR}[C_{i}][R](K_{OR}+[R])$$

The above equations simplify to:

$$\begin{matrix} (k_{dA}K_{OA^{'}}-\frac{k_{se}}{k_{C}}k_{dA}[C_{e}])[R]^{3} \\ +(k_{dA}K_{OA^{'}}K_{OR}-\alpha_{A}+k_{dA}K_{OA^{'}}K_{OA}+\frac{k_{dA}\alpha_{R}}{k_{C}}-\frac{k_{se}}{k_{C}}k_{dA}[C_{e}](K_{OR}+K_{OA}))[R]^{2} \\ +(-\frac{k_{se}}{k_{C}}k_{dA}[C_{e}]K_{OA}K_{OR}-\alpha_{A}K_{OR}+k_{dA}K_{OR}K_{OA^{'}}K_{OA}+\frac{k_{dA}\alpha_{R}}{\alpha_{CR}k_{C}}(\alpha_{CR}K_{OA}+k_{se}))[R]+\frac{k_{dA}\alpha_{R}k_{se}K_{OA}}{\alpha_{CR}k_{C}}=0 \end{matrix}$$

The product of three roots for the above cubic equation is given by $\frac{\alpha_{R}k_{se}K_{OA}}{\alpha_{CR}k_{C}}/(\frac{k_{se}}{k_{C}}[C_{e}]-K_{OA^{'}})$ and the sum of roots is given by $((K_{OA^{'}}-\frac{k_{se}}{k_{C}}[C_{e}])(K_{OR}+K_{OA})-\alpha_{A}+\frac{\alpha_{R}}{k_{C}})/(\frac{k_{se}}{k_{C}}[C_{e}]-K_{OA^{'}})$. The above equation will have two positive roots (and one negative root) of [*R*] if the product is negative and the sum of roots is positive i.e. if $K_{OA^{'}}-\frac{k_{se}[C_{e}]}{k_{C}}>0$ (or $[C_{e}]<\frac{K_{OA^{'}}k_{C}}{k_{se}}$) and $(K_{OA^{'}}-\frac{k_{se}}{k_{C}}[C_{e}])(K_{OR}+K_{OA})+\frac{\alpha_{R}}{k_{C}}<\alpha_{A}$.

For $[C_{e}]>\frac{K_{OA^{'}}k_{C}}{k_{se}}$the product of roots will be positive and the sum will be negative i.e. there is one positive root of [*R*]. For physiologically feasible steady states, concentration of all other species should also be positive. From equations 1.15 and 1.16 $[A]=\frac{\alpha_{A}}{k_{dA}(K_{OA}+[R])}-K_{OA^{'}}$ and $[C_{i}]=\frac{k_{C}.[A]+k_{se}.[C_{e}]}{\alpha_{CR}[R]+k_{se}}$. Therefore, while $[C_{i}]$ is always positive, [*A*] will be positive only for $[R]<\frac{\alpha_{A}}{k_{dA}K_{OA^{'}}}-K_{OA}$. Note, that this is also the stability condition for the fixed state corresponding to [*A*]=0.

Thus, for $[A]\neq0$ the system will have two positive fixed points if $[C_{e}]<\frac{K_{OA^{'}}k_{C}}{k_{se}}$ and $[R]<\frac{\alpha_{A}}{k_{dA}K_{OA^{'}}}-K_{OA}$. Beyond [*C_e_*] values where $[R]>\frac{\alpha_{A}}{k_{dA}K_{OA^{'}}}-K_{OA}$there is only one fixed state.

An analytical condition for the stability of the roots is difficult to determine. A stable steady state requires all the eigen values of the Jacobian matrix to be negative. The trace of the Jacobian matrix for equation 1.18 is always negative. The determinant will be negative if the following condition is satisfied:

$$\begin{matrix} k_{da}k_{dA}^{2}(K_{OA}+[R])(k_{se}+\alpha[R])(\frac{\alpha_{R}}{(K_{OR}+[R])^{2}}+\frac{\alpha(k_{se}[C_{e}]+k_{C}[A])}{k_{se}+\alpha[R]}) \\ <\alpha k_{mA}K_{OA}k_{pA}[R](-\frac{k_{C}k_{dA}}{(K_{OA}+[R])}-\frac{\alpha k_{da}k_{dA}^{2}(K_{OA}+[R])(k_{se}[C_{e}]+k_{C}[A])}{k_{mA}K_{OA}k_{pA}(k_{se}+\alpha[R])}) \end{matrix}$$

Negative trace and determinant is a necessary but not sufficient condition for all eigen values to be negative.

To summarize, the simplified ScbA-ScbR system represented by equations 1.1 - 1.6 will have three positive fixed points for increasing [*Ce*] until the transition point that satisfies $[R]=\frac{\alpha_{A}}{k_{dA}K_{OA^{'}}}-K_{OA}$. One fixed point corresponds to [A]=0, whereas the other two are roots of equation 1.18 where $[A]\neq0$. For [*Ce*] values where $[R]>\frac{\alpha_{A}}{k_{dA}K_{OA^{'}}}-K_{OA}$, there are two fixed points only one of which can be stable.

Figure S5A illustrates the steady states for a set of parameter values that satisfy the above conditions. It was observed that one of the two roots corresponding to lower value of [*R*] was stable and the other unstable. This stable steady state corresponding to a low [*R*] results in low degree of suppression of *scbA*. This in turn leads to high values of *A*, *C_i_*, *C_e_* and *CR* complex, which represents the ON state for expression of *cpk* cluster genes. In this case, the production of ScbR is balanced by the formation of SCB1-ScbR (*CR*) complex.

Note that the basal transcription rate of *scbA* was neglected in this analysis. If the basal rate is accounted for, there is only one change in the steady state plot. The unstable steady state corresponding to [*A*] = 0 disappears beyond the transition point. The rest of the steady states are not affected. The steady state diagram is shown in Figure S5B.
